# Interrupting Sitting Time in Postmenopausal Women: Protocol for the Rise for Health Randomized Controlled Trial

**DOI:** 10.2196/28684

**Published:** 2021-05-13

**Authors:** Sheri J Hartman, Lindsay W Dillon, Andrea Z LaCroix, Loki Natarajan, Dorothy D Sears, Neville Owen, David W Dunstan, James F Sallis, Simon Schenk, Matthew Allison, Michelle Takemoto, Alexandra M Herweck, Bao Nguyen, Dori E Rosenberg

**Affiliations:** 1 Hebert Wertheim School of Public Health University of California, San Diego La Jolla, CA United States; 2 Moores Cancer Center University of California, San Diego La Jolla, CA United States; 3 Department of Medicine University of California, San Diego La Jolla, CA United States; 4 College of Health Solutions Arizona State University Phoenix, AZ United States; 5 Department of Family Medicine University of California, San Diego La Jolla, CA United States; 6 Baker Heart and Diabetes Institute Melbourne Australia; 7 Centre for Urban Transitions Swinburne University Melbourne Australia; 8 Mary MacKillop Institute for Health Research Australian Catholic University Melbourne Australia; 9 Department of Orthopaedic Surgery University of California, San Diego La Jolla, CA United States; 10 ServiceNow Santa Clara, CA United States; 11 School of Medicine University of California, San Diego La Jolla, CA United States; 12 Kaiser Permanente Washington Health Research Institute Seattle, WA United States

**Keywords:** sedentary behavior, cardiometabolic health, older adults, physical function, cognitive function, biomarkers

## Abstract

**Background:**

Many older adults spend the majority of their waking hours sitting, which increases their risk of chronic diseases. Given the challenges that many older adults face when engaging in moderate-to-vigorous physical activity, understanding the health benefits of decreasing sitting time and increasing the number of sit-to-stand transitions is needed to address this growing public health concern.

**Objective:**

The aim of this 3-arm randomized controlled trial is to investigate how changes in sitting time and brief sit-to-stand transitions impact biomarkers of healthy aging and physical, emotional, and cognitive functioning compared with a healthy attention control arm.

**Methods:**

Sedentary and postmenopausal women (N=405) will be recruited and randomly assigned to 1 of the 3 study conditions for 3 months: healthy living attention control (Healthy Living), reduce sitting time (Reduce Sitting), and increase sit-to-stand transitions (Increase Transitions). Assessments conducted at baseline and 3 months included fasting blood draw, blood pressure, anthropometric measurements, physical functioning, cognitive testing, and 7 days of a thigh-worn accelerometer (activPAL) and a hip-worn accelerometer (ActiGraph). Blood-based biomarkers of healthy aging included those associated with glycemic control (glycated hemoglobin, fasting plasma insulin and glucose, and homeostatic model assessment of insulin resistance).

**Results:**

Recruitment began in May 2018. The intervention is ongoing, with data collection expected to continue through the end of 2022.

**Conclusions:**

The Rise for Health study is designed to test whether 2 different approaches to interrupting sitting time can improve healthy aging in postmenopausal women. Results from this study may inform the development of sedentary behavior guidelines and interventions to reduce sitting time in older adults.

**Trial Registration:**

ClinicalTrials.gov NCT03473145; https://clinicaltrials.gov/ct2/show/NCT03473145

**International Registered Report Identifier (IRRID):**

DERR1-10.2196/28684

## Introduction

### Background

Many older adults find it challenging to engage in health-enhancing physical activity (PA) and can spend up to 11 hours a day sitting [1]. Greater amounts of sedentary time, including time spent sitting and lack of PA, are associated with an increased risk of type 2 diabetes; all-cause mortality; cardiovascular disease; higher insulin; hypertension; and, specifically in older adults, poorer physical functioning [2-11]. Older women, in particular, are at an increased risk of chronic diseases and disabilities [3]. Furthermore, older women require more health care than other segments of the population and account for approximately 14% of all outpatient physician visits and 22% of hospitalizations, although they represent only 7% of the US population [12]. With the high use of health care, feasible behavior change interventions are needed to improve health and physical function.

Despite decades of public health efforts to encourage a minimum of 30 minutes per day of moderate-to-vigorous physical activity (MVPA), which has many positive health effects in older adults [13], only 3% to 6% of older adults meet public health guidelines for MVPA [14,15]. Although there have been some successful PA interventions for older adults (eg, Lifestyle Independence for Elders [16]), social and environmental conditions, functional challenges, and disease factors may limit the capacity of many older adults to engage in MVPA [17]. One promising alternative strategy to improve health is to interrupt sitting behaviors. This may be a more viable behavior change goal for many older adults because it does not involve high-intensity or high-impact activity and can be done anywhere and at any time. It is also possible that intervening on sitting behaviors, for example, by reducing the time spent sitting or increasing sit-to-stand transitions, could be the first step toward a more active lifestyle [18]. Changing sedentary behavior by reducing the time spent sitting has yielded favorable changes in blood pressure, glucose control, insulin sensitivity, and waist circumference [19]. Data from randomized controlled trials (RCTs) are needed to determine if changes in sedentary time lead to health improvements in older adults outside of worksite settings.

Several feasibility studies have indicated that time spent being sedentary can be reduced in older adults by 30-60 minutes per day [20-24]. Existing studies with older adults generally have small sample sizes, poor methodological quality, and relatively short durations [25]. The majority of previous studies have focused on reducing sitting time and, therefore, have not found appreciable improvements in sit-to-stand transitions. However, sit-to-stand transitions have been shown to increase postural blood flow and positively impact insulin and homeostatic model assessment of insulin resistance (HOMA-IR) [8,26-29]. There is a need to better understand how changing these distinct aspects of sedentary time, sitting less and transitioning more, may impact biological, physical, cognitive, and emotional health.

### Objectives

The Rise for Health trial seeks to address gaps in the literature and is based on a pilot study conducted by our team. In a 2-week study, older adults were randomized to either reducing their sitting time or increasing their sit-to-stand transitions [21]. This preliminary study found that those in the reducing sitting arm decreased their sitting by 130 minutes without changing their sit-to-stand transitions, and participants in the increasing sit-to-stand transition arm increased their transitions by about 13 per day but did not change their sitting time [21]. These findings highlight the distinct nature of these 2 aspects of sedentary time and the need to understand how changing each affects health. Rise for Health builds on the findings of this study by conducting a fully powered 3-month intervention. The ultimate goal of Rise to Health is to determine whether reducing the overall time spent sitting or increasing brief sit-to-stand transitions, as measured with a thigh-worn accelerometer (activPAL; PAL Technologies), results in changes in health outcomes, including glucose control, blood pressure, and physical and cognitive function, in older, overweight women.

## Methods

### Overview

Rise for Health is a 3-arm RCT of 2 interventions that address changing sitting in overweight, postmenopausal women over a 3-month period, compared with an attention control condition. This study is one of the three projects within the National Institute on Aging Program Grant named *Sedentary Time and Aging Mortality and Physical Function* (STAR). The program grant proposes a paradigm shift away from energy expenditure as the primary mechanism for improving health outcomes to investigate potentially feasible behaviors such as brief sit-to-stand transitions that expend little energy but engage muscles, improve postural blood flow, and may impact physical functioning in older adults. The STAR program includes 3 projects and 2 cores for studying postmenopausal women at risk of chronic diseases. In addition to the RCT described here (project 2), STAR includes a 3-condition randomized crossover laboratory trial of strategies to interrupt sitting time (n=78; project 1) and a study that optimizes new computational techniques for objectively measuring sedentary behavior to apply to existing prospective hip-worn accelerometer data from the Objective Physical Activity and Cardiovascular Disease Health in Older Women (OPACH) cohort of the Women Health Initiative (N>6000, project 3). All projects are investigating the consequences of sitting and brief sit-to-stand transitions on the mechanisms of healthy aging, including glucose regulation and endothelial functioning. The STAR program will provide a comprehensive evidence base that can inform public health guidelines on sitting behaviors and healthy aging.

### Study Objectives

Rise for Health will examine 3-month changes in biomarkers of healthy aging and physical, emotional, and cognitive functioning across a 3-arm randomized trial: (1) healthy living attention control (Healthy Living), (2) reduce sitting time (Reduce Sitting), and (3) increase sit-to-stand transitions (Increase Transitions). The study aims to enroll 405 postmenopausal women into the 3-month trial. The primary aim is to compare changes in glucoregulatory biomarkers (fasting plasma insulin and glucose, HbA_1c_, and HOMA-IR) and blood pressure over 3 months for the 2 intervention arms compared with the attention control condition. We hypothesize that, compared with those allocated to the attention control arm, participants in the 2 intervention arms will have greater improvements in glycemic control and blood pressure. In addition, we will evaluate the dose-response effects of sitting behavior changes on glucoregulatory biomarkers and blood pressure. We hypothesize that greater improvements in the target behavior will be associated with greater improvements in glucoregulatory biomarkers and blood pressure. The secondary aims of the study are to examine the effects of the intervention on physical, emotional, and cognitive functioning. This project will also explore (1) the modifying effects of age on the relationship between the 3 conditions and primary and secondary outcomes, (2) the psychosocial and mediators and moderators of changes in sitting behaviors, and (3) the differences in outcomes between the 2 sitting interruption intervention arms. The purpose of this paper is to describe the study protocol of Rise for Health, a 3-arm randomized trial, to assess ways of interrupting sitting in postmenopausal women.

The institutional review board of the University California San Diego (UC San Diego) approved all study procedures, and all participants will provide written informed consent. Study recruitment, participant safety, and progress are reviewed semiannually by an external independent data safety monitoring board appointed by the National Institutes of Health (NIH).

### Participants

#### Eligibility

To be eligible, participants must be female and meet the following inclusion criteria: currently 55 years or older; sit for more than 7 hours on a majority of device-measured days, as assessed by activPAL (as given in the *Screening Visit* section); do 70 or fewer sit-to-stand transitions on a majority of device-measured days, as assessed by activPAL; no health conditions that would inhibit standing; able to read and write fluently in English; postmenopausal, defined as no menstrual period in the last 12 months; BMI≥25 kg/m^2^ and <45 kg/m^2^; able to walk, stand, and perform sit-to-stand transitions without a high risk of falling, determined by the Short Physical Performance Battery (SPPB); and able to travel to study visits. Exclusion criteria include the use of insulin, uncontrolled diabetes defined as HbA_1c_>10%, uncontrolled blood pressure defined as systolic blood pressure>180 or diastolic blood pressure>110, and participation in another research study or program that would impact the outcomes of this study.

#### Recruitment and Screening

The primary methods of recruitment are contacting UC San Diego patients identified through electronic health records and contacting women in San Diego through marketing lists. Women are mailed a letter and a flyer to explain the study. Prospective participants are informed that the study staff would contact them or they could call to opt out. Additional recruitment methods include advertisements on social media platforms, such as Facebook and Instagram, flyers and listserv postings, and ResearchMatch [30].

Trained recruiters describe the study activities and conduct phone eligibility screening with potential participants. After phone screening, eligible women are scheduled for an in-person screening visit at UC San Diego.

#### Screening Visit

At the initial visit, study requirements are reviewed and signed informed consent is obtained. Next, participants complete a medical history questionnaire and self-report their current medication and supplement use to confirm they do not have a medical condition that would inhibit standing or sit-to-stand transitions and do not use insulin. Measurements of height, weight, and blood pressure are taken to screen for BMI and blood pressure. Hip and waist circumference measurements are also recorded. Participants perform SPPB to assess physical functioning and the ability to safely stand and perform sit-to-stand transitions. If all the preliminary screening criteria are met, participants complete a battery of cognitive tests and questionnaires on self-reported demographics and PA. Participants are given an activPAL, a thigh-worn accelerometer that objectively measures sitting time and number of sit-to-stand transitions, and an ActiGraph GT3X+ (ActiGraph, LLC) accelerometer, a waist-worn device that objectively measures minutes of PA. Participants are shown how to attach activPAL using waterproof Tegaderm dressing so that it does not have to be removed, and replacement waterproof dressing is provided. Participants are asked to wear these devices for 24 hours continuously, except they are asked to remove the ActiGraph GT3X+ accelerometer before bathing or swimming for the next 7 days.

#### Baseline Visit

Participants return to the clinic after at least seven days of wearing the two devices. Data from activPAL are screened for sitting time (more than 7 hours on a majority of measured days) and sit-to-stand transitions (70 or fewer transitions per day on a majority of measured days) to confirm eligibility. Participants provide fasting blood samples via a finger prick to screen for HbA_1c_. Those who pass these final eligibility criteria then have a venous blood draw taken by a certified phlebotomist and complete additional questionnaires. Participants receive a total of US $35 for completing baseline measures. After all baseline assessments are complete, the participants are then randomized.

Randomization is stratified by BMI (overweight vs obese) and employment status (full-time vs nonfull-time employment). A computerized randomization scheme was created using the STAR program grant Biostatistics Core, using a random number generator. A stratified permuted block design is used in this study. After randomization, the participants review the expectations and requirements of their study group assignment (details given in the *3-Month Intervention* section). Data collectors and the principal investigator are blinded to the study group assignment.

#### 3-Month Final Visit

Before their final visit, participants are mailed the accelerometer and activPAL and asked to wear both devices continuously for 7 days before the visit. At this visit, participants repeat the same measures collected at the screening and baseline visits, including blood draw, anthropometric measures, battery of cognitive tests, SPPB, and questionnaires. Participants receive up to US $110 for completing the final measures.

### 3-Month Intervention (Rise for Health)

#### Reduce Sitting and Increase Sit-to-Stand Transitions

The primary goal of the respective intervention arms is to interrupt the current sitting patterns by targeting 2 specific behavior changes: reducing the amount of time spent sitting (Reduce Sitting) or by increasing the number of sit-to-stand transitions each day (Increase Transitions). Both interventions, Reduce Sitting and Increase Transitions, use habit formation [31-34], social cognitive theory [35], and motivational interviewing techniques to support behavior change. See Figure S1 in Multimedia Appendix 1 for the outline of the intervention topics and schedule of activities.

##### Health Coaching Sessions for Reduce Sitting and Increase Transitions

Participants in both intervention arms receive 5 in-person, individual coaching sessions (weeks 1, 2, 3, 4, and 8) and 2 individual phone coaching sessions (weeks 6 and 11) over the course of the 12-week program. Participants are asked to wear activPAL on their thigh continuously for the first 4 weeks of the study and then again during weeks 7 and 8 to help with self-monitoring, goal setting, and personalized feedback.

**Figure 1:**
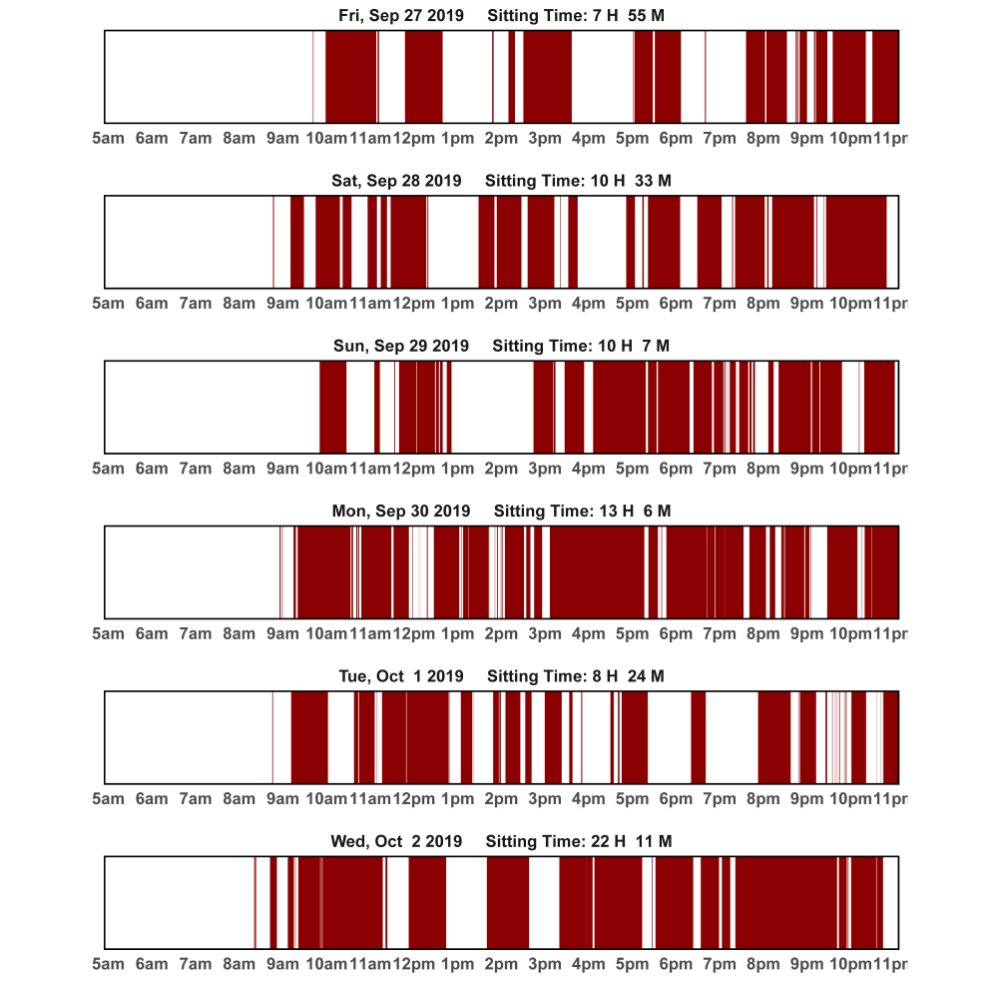
Sample feedback graph for the Reduce Sitting group: day-level activPAL data; red indicates where sitting occurred.

**Figure 2:**
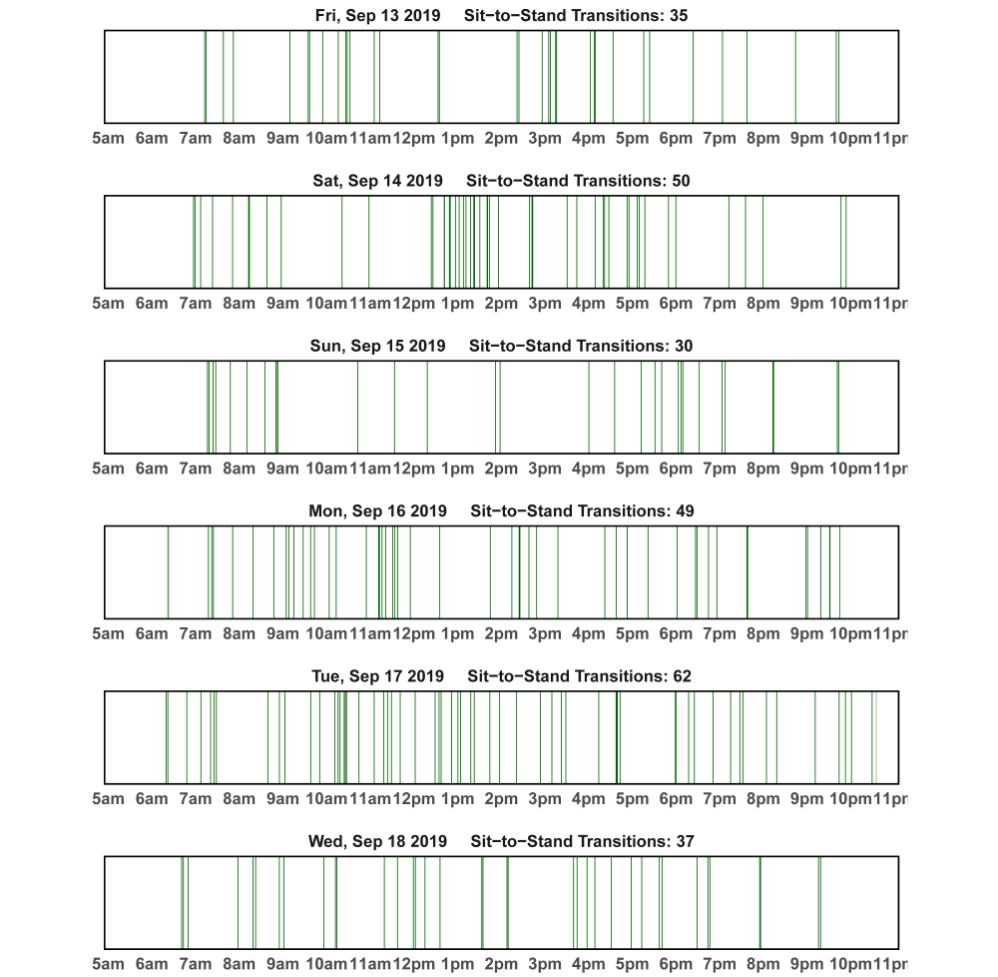
Sample feedback graph for sit-to-stand transitions: day-level activPAL data; green indicates where a sit-to-stand transition occurred.

During the first 60-minute in-person session, the health coach provides an overview of the intervention and gives participants a binder of printed educational materials and safety tips, printed action plan forms, and tracking logs. First, participants are asked to share their motivation to join the study. Next, they review reasons for sitting, dangers of prolonged sitting, and benefits of breaking up sitting. Tips for safely reducing sitting or increasing sit-to-stand transitions based on group assignments are reviewed. The health coach models the target behavior by having participants in the Reduce Sitting arm practice standing for up for 5 minutes in the middle of the session, and participants in the Increase Transitions arm perform 3 brief sit-to-stand transitions in a row, holding each for 5 seconds, twice during the session. Participants are then given a wrist-worn activity band that is programmed to prompt sitting breaks based on group assignment (refer to the *Toolbox* section). Participants are shown graphs from activPAL data, either of time spent sitting or sit-to-stand transitions, depending on the group assignment (Figures 1 and 2). The health coach and participants use the graphs to (1) review a typical day and identify times during the day when participants may be able to take sitting breaks or perform sit-to-stand transitions, (2) set behavior change goals for the next week, and (3) create a specific action plan. Participants complete an action plan by identifying the specific strategy, location, days of the week, time of day, and tailored goals for the participant (number of minutes to reduce sitting by or number of transitions to complete). Participants brainstorm potential obstacles to following the action plan and solutions for overcoming each obstacle. Using motivational interviewing techniques, confidence is assessed and supported using a 0 (very low confidence) to 10 (very high confidence) scale (*ruler*). To further support behavior change, participants are provided with several tools they can choose to use. As people vary in their preferences and needs, a toolbox approach allows participants to select tools they want to try (refer to the *Toolbox* section). At the end of the session, participants are given an activPAL to wear for the next 7 days and bring in the following week so that they can review their behavior with their health coach. If the participant provides consent, the health coach sessions are audio recorded for quality checks and training.

**Figure 3:**
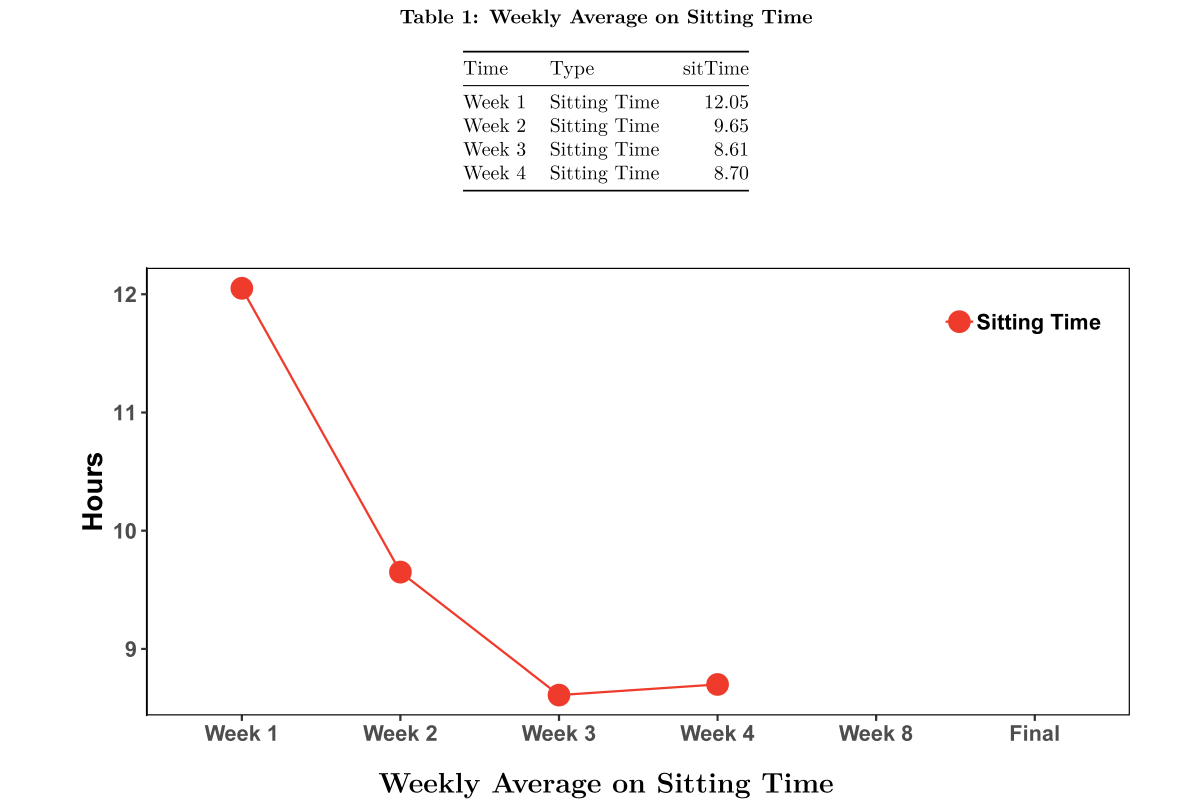
Sample feedback graph during intervention for the Reduce Sitting group: week-level activPAL data; average sitting time.

**Figure 4:**
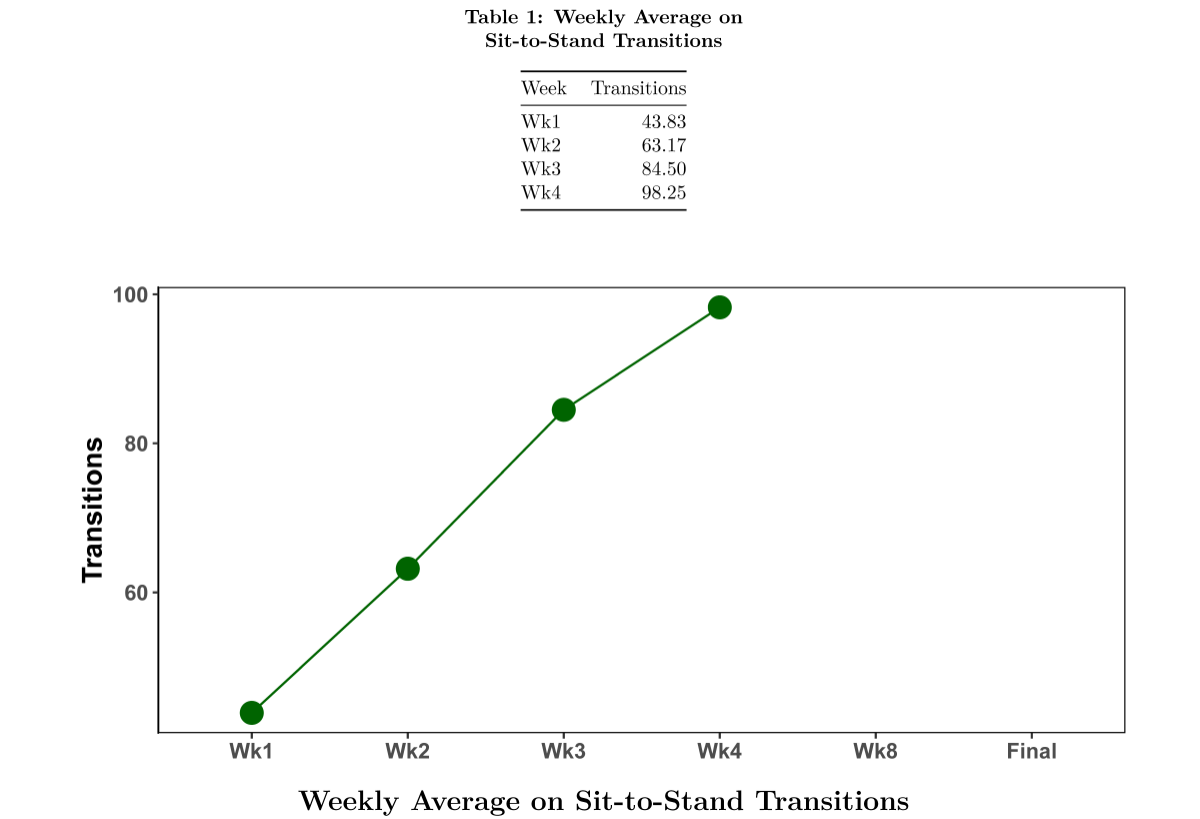
Sample feedback graph during intervention for Sit-to-Stand Transition Group: week-level activPAL data; average sit-to-stand transitions.

At each subsequent coaching session, the health coach starts by assessing the adverse events that may have occurred since the last session. The health coach and the participant then model the respective behavior, gradually increasing the number of minutes of standing or number of transitions each week until week 4, when standing time may be up to 10 minutes and the number of transitions at the beginning of the session may be 5. The health coach reviews any key topics from the previous session before introducing the new behavior change topic (Figure S1 in Multimedia Appendix 1). When worn, the previous week’s daily activPAL data are reviewed using daily graphs of data (Figures 1 and 2), as described above for week 1. Graphs of weekly data are shown to discuss progress toward the target behavior (Figures 3 and 4). At each session, the health coach supports the updating of personalized goals and action plans. There is a second break to model the target behavior before completing the action plan. Discussions on barriers and solutions and the confidence to meet the goal are addressed. Weeks 2, 3, 4, and 8 sessions are carried out in person and last approximately 60 minutes each. Weeks 6 and 11 sessions are carried out over the phone and last approximately 30 minutes each. Participants are given activPAL to wear in weeks 1, 2, 3, 4, 8, and 9.

##### Intervention Toolbox

Participants are provided with a toolbox of options to help with their personalized behavior goals. Participants can choose different tools at each in-person session and can keep tools for the duration of the study or trade them in for new tools as desired. All participants are encouraged to use a wrist-worn device (eg, Watchminder) that provides reminders to engage in the behavior. The wrist-worn device is programmed to vibrate and display the message *Rise Up* periodically from 8 AM to 9 PM every day; however, participants have the option to change these times to match their personalized action plan. For participants in the Reduce Sitting arm, the reminder is set to once an hour. For participants in the Increase Transitions arm, the reminder is set to every 20 minutes. Other tools offered to help prompt behavior change include lists of computers, tablets, and phone apps that prompt sitting breaks; egg timers; and visual reminders (cue cards and a study-branded bracelet). Participants in the Reduce Sitting arm have the option of a standing desk to use at home or work starting in week 2, and participants in the Increase Transitions arm have the option of a tally counter to count transitions throughout the day.

#### Healthy Living

Participants in the healthy living attention control (Healthy Living) condition receive an equal number of contacts as those in the 2 intervention arms. The first session (week 1) is delivered in-person, whereas the remaining sessions (weeks 2, 3, 4, 6, 8, and 11) are delivered over the phone. During the first session, the health coach provides an overview of the study group and gives the participant a folder that includes information, worksheets, and resources on various healthy aging topics that may be discussed throughout the study. A new healthy aging topic is discussed at each session, with sleep and aging as the first topic. For all future phone sessions (weeks 2, 3, 4, 6, 8, and 11), the participant chooses which healthy aging topic to discuss. Example topics include safe driving, stress reduction, and healthy bones (Figure S1 in Multimedia Appendix 1). At each session, the health coach first provides an introduction to the selected topic. The health coach then reviews the learning objectives for the session, provides additional information about the health topic, and prompts the participant to complete the topic’s worksheet. The health coach and participant then set the goal of the participant’s choice related to the topic for that session and develop a related action plan. Using motivational interviewing techniques, confidence is assessed and supported using a 0-10 ruler question. At the end of each session, the health coach summarizes the session and confirms the date and time of the next session. The initial in-person session lasts 60 minutes, and subsequent phone sessions last 30 minutes.

### COVID-19 Considerations

Owing to the COVID-19 pandemic, temporary pauses in enrollment of new study participants and in-person sessions occurred and may continue to occur consistent with UC San Diego research policies and San Diego County health orders. To minimize data loss during the pause of in-person visits, enrolled participants whose final 3-month assessment visits are scheduled during office closures will be asked to complete measures remotely. For remote measures, the participants are mailed an automated blood pressure machine and a measuring tape. A Zoom videoconferencing session is scheduled to obtain the blood pressure, waist and hip circumferences, balance tests, chair raises (as part of the SPPB), and NIH Toolbox measures (oral symbol digit test and the list sorting working memory test) using the NIH Toolbox recommendations for remote delivery [36]. Participants are mailed activPAL and ActiGraph to objectively measure sedentary time and sit-to-stand transitions per protocol on the due date. Survey measures through REDCap (Research Electronic Data Capture) [37-39] are emailed to participants on the due date. Participants are offered extended phone coaching with continued behavior change support until they are able to complete a blood draw either via mobile phlebotomy that goes to their home or upon our ability to conduct in-person assessments. Additional measures of anxiety (PROMIS Bank v.10–Anxiety and Cognitive and Affective Mindfulness Scale–Revised) have been added to better understand how anxiety and stress during this period relate to behavior change.

The study protocols may continue to shift to support remote delivery and to respond to the changing requirements related to the COVID-19 pandemic to maintain safety for participants and study staff.

### Measures and Outcomes

#### Primary Outcomes

##### Glucose Regulation and Blood Pressure

The primary outcomes are glucose regulation and blood pressure assessed at baseline and at 3 months. Glucose regulation will be assessed by fasting plasma insulin and glucose, HbA_1c_, and HOMA-IR. Fasting blood (45 mL) is collected in EDTA and Li-Heparin vacutainers. Frozen processed samples will be stored in locked freezers at −80 C. Plasma and whole blood samples will be used for glucose and HbA_1c_ assays, and all analyses will include normalization and quality control standards. HbA_1c_ is measured in whole blood in real time (DCA Vantage, Siemens). After blood sample collection is complete, fasting plasma glucose will be measured using the standard glucose oxidase method (YSI Bioanalyzer) and fasting plasma insulin will be measured using an immunoassay kit (Meso Scale Discovery, catalog #K151BZC). Standard curve samples and quality control replicates (minimum of 2 per plate) will be run on each assay plate. Linear dilution and spike-in controls will also be included in each assay run. Additional aliquots of samples will be stored at −80 °C should repeat analyses be required and for future ancillary analyses. Blood pressure is measured using the Dinamap or Accutor 7 or Dinamap V100 blood pressure monitor after participants have rested while seated for at least 5 minutes. Measures are taken at least three times, and the mean of the second and third readings will be calculated.

##### Objective Measure of Sitting Time and Sit-to-Stand Transitions

Measures used to examine the dose-response effects of behavioral changes on glucoregulatory biomarkers and blood pressure are activPAL and ActiGraph GT3X+. For 7 days before the baseline and 7 days before the 3-month final visit, participants are asked to wear the 2 devices for 24 hours a day. activPAL is a small and lightweight accelerometer worn on the anterior aspect of the thigh that produces a signal related to thigh inclination, which is used to estimate the time spent in different body postures (sitting and standing) and the number of sit-to-stand transitions [40]. activPAL has demonstrated good reliability and validity [41-43]. activPAL data will be downloaded using activPAL Professional Research Edition software package using the 15-second Epoch. To filter nighttime sleeping, participants are asked to keep a sleep log each night they wear the activPAL. ActiGraph GT3X+ is a small device attached to a belt, positioned over the right hip. The ActiGraph collects data on 3 axes at 30 Hz to estimate the minutes spent in sedentary, light, moderate, and vigorous activity using calibration thresholds. ActiGraph has been validated against heart rate telemetry [44] and total energy expenditure [45,46]. Nonwear time will be classified using Choi algorithm with 90 consecutive zero counts on the x-axis [47]. We will use the newly validated OPACH cut points to assess moderate PA [48]. In addition, data will be provided to the Biostatistics Core and project 3 for further analysis of bouts, time of day, and clustering with sleep and to validate the machine-learned algorithms.

#### Secondary Outcomes

Secondary outcomes focus on physical, emotional, and cognitive function and will be measured at baseline and at 3 months. Objective lower extremity functioning will be measured using the SPPB to measure balance, gait speed (4 meters course), and chair stand [49]. Self-report physical functioning will be measured using 6 items from the Activities of Daily Living Survey, which focuses on the ability to perform basic tasks of everyday life, such as eating, bathing, or dressing with or without assistance [50,51]. Physical and emotional functioning will be measured using the Physical Functioning Survey [52]. It comprises 36 items that assess 8 health concepts: physical functioning, role limitations caused by physical health problems, role limitations caused by emotional problems, social functioning, emotional well-being, energy or fatigue, pain, and general health perceptions. Physical Functioning Survey has been found to be reliable, valid, and responsive for a variety of medical diagnoses. Depressive symptoms will be measured using the validated 10-item Center for Epidemiologic Studies Depression Scale short form [53]. These outcome measures were selected for consistency with the outcome measures used in the cohort for project 3 of the STAR program.

Objective cognitive functioning will be measured using the Dimensional Change Card Sort, List Sorting Working Memory, and Oral Symbol Digit from the NIH Toolkit [54,55]. These tests assess executive function, memory, and processing speed, respectively. These 3 tests take about 17 minutes to administer via an iPad (Apple Inc) app and have been validated and normed in individuals aged 3-85 years [54,55]. Self-report cognitive functioning is assessed using the 8-item Cognitive Function Questionnaire from the Patient-Reported Outcomes Measurement Information System (PROMIS) [56].

#### Additional Measures

As psychosocial factors may mediate and moderate sedentary behavior changes, these domains are also measured. Self-reported perceptions of sleep quality, sleep depth, and restoration associated with sleep are measured using the NIH PROMIS Sleep Disturbance 8a Short Form [57]. Self-reported barriers and benefits related to sitting are assessed on a 9-item, 5-point Likert scale assessing the mental and physical factors that affect their ability to sit adapted from a scale for PA [58]. Participants are asked to rate their confidence in performing the targeted sedentary behaviors (sitting reduction and increase in sit-to-stand transitions) on a 2-item confidence scale, which is modeled after a PA self-efficacy scale [59]. Self-reported PA is measured at baseline using the Community Healthy Activities Model Program for Seniors Physical Activity Questionnaire for Older Adults, which asks about weekly frequency and duration of a variety of lifestyle physical activities that are meaningful and appropriate for older adults [60]. Community Healthy Activities Model Program for Seniors was selected for measurement consistency across all the STAR projects. Anxiety will be assessed using the NIH PROMIS computer-adaptive version of their anxiety measure [61]. Cognitive and Affective Mindfulness Scale–Revised is a 12-item measure that captures mindful approaches to thoughts and feelings [62]. Pain will be measured using the PROMIS pain interference NIH PROMIS pain interference and intensity short forms and modified items recommended by the NIH Task Force for Research Standards for Chronic Low Back Pain [63,64]. Additional measures that were adapted from validated measures to specifically address sedentary behavior include the Self-Report Behavioral Automaticity Index [65] and habits and perceptions [66]. A complete list of measures is given in Textbox 1.

**Rise for Health study measures. Measures are collected at baseline and at 3 months except where noted.**

**Primary outcomes**

- Glucose regulation biomarkers
  - Glucose, insulin, and HbA_1c_ measurements. Glucose and insulin will be assessed individually and in combination to measure insulin sensitivity
- Blood pressure
  - Mean of the second and third readings of 3 measures

**Objective sedentary and physical activity**

- Sedentary behavior
  - 7-day, 24-hour, thigh-worn activPAL
- Physical activity
  - 7-day, 24-hour, hip-worn ActiGraph GT3X+

**Secondary outcomes**

- Physical function—objective
  - Short Physical Performance Battery (balance, gait speed, and chair stands) [49]
- Physical function—self-report
  - Activities of Daily Living [50] and Medical Outcome Study 36-item short form health survey [52]
- Depressive symptoms
  - Centers for Epidemiologic Studies Depression Scale short form [53]
- Cognitive function—objective
  - National Institutes of Health (NIH) Toolbox Cognition Tests: Dimensional Change Card Sort, List Sorting, and Oral Symbol Digit [54,55]
- Cognitive function—self-report
  - Patient-Reported Outcomes Measurement Information System (PROMIS) Cognitive Function Measure [56]

**Other relevant measures**

- Height and weight
  - Digital scale and stadiometer (height baseline only)
- Waist and hip circumference
  - Assessed in centimeters
- Demographics (baseline only)
  - Age, education, income, race, and marital status with standard surveys
- Medical history—self-report
  - Medical history, recent medical history, and medications
- Psychosocial measures
  - NIH PROMIS Anxiety [61], Cognitive and Affective Mindfulness Scale–Revised [62], benefits and barriers [58], self-efficacy [59], Self-Report Behavioral Automaticity Index [65], habits, and perceptions [66]
- Physical activity—self-report
  - Community Healthy Activities Model Program for Seniors Physical Activity Questionnaire for Older Adults (baseline only) [60]
- Sleep—self-report
  - NIH PROMIS Sleep Disturbance 8a Short Form [57]
- Pain—self-report
  - NIH PROMIS Pain Interference and Pain Intensity Short Form [63,64]

#### Statistical Analysis and Sample Size Considerations

We aim to recruit 405 participants for the study to ensure 80% power to detect meaningful improvements in glucose regulation after accounting for a 10% dropout or missing data (eg, unassayable sample) rate. Given the multiple correlated biomarker outcomes, we will create a composite outcome derived as a sum of z-scores of the (possibly transformed) markers. A few previous interventions aimed at decreasing sedentary time in adults or increasing standing observed effect sizes between 0.39 and 0.49 on fasting insulin. Thus, we based sample size estimates on an assumed effect size of 0.4 at 3 months. We set the significance level α=.025 for 2 comparisons (each of the sitting interruption interventions compared with the control). Under these assumptions, we would need 121 subjects per arm, based on a 2-sided (2-tailed), 2-sample *t* test to detect an effect size of 0.4 with 80% power between the 2 sitting interruption arms compared with the control arm. To translate these effect sizes to biomarker values, we used preliminary data from our OPACH study of approximately 6000 postmenopausal women who had a mean of 98.3 mg/dL (SD 27.7) for fasting glucose and a log-transformed mean of 4.17 pmol/L (SD 0.77) for insulin. Assuming the same SDs as in the OPACH study, we have 80% power to detect the average changes of 11.1 mg/dL for glucose (SD 27.7) and 0.31 pmol/L (SD 0.77) for (log)insulin in the intervention arms versus no change in controls. Recruitment projections before the commencement of the study indicated 405 participants to be feasible. Recruitment will stop when the study reaches either the required sample size or the maximum number of participants who can be recruited in the study’s allotted recruitment timeframe, while complying with the unforeseeable restrictions and conditions related to the COVID-19 pandemic.

To check if randomization achieved balance on key covariates, study groups will be compared based on baseline characteristics using the analysis of variance (for continuous variables) and chi-square (for categorical) variables. Variables that are not balanced across study groups will be adjusted for in subsequent analyses. For intervention effect comparisons, we will apply a mixed effects analysis approach in which all available assessments on an individual can be included in the model. Gaussian link functions will be used for continuous outcomes (eg, biomarkers), and a binomial or loglinear link will be used for binary or categorical outcomes. Biomarker outcomes will be transformed as needed to better approximate the Gaussian distributions for model residuals. The primary superiority analysis comparing the Reducing Sitting and Increasing Transitions interventions with controls will use the intent-to-treat principle. We will include adherence measures to explore intervention differences by compliance level.

#### Analysis Plans

##### RCT Analysis Plan for Primary and Secondary Outcomes

We will use mixed effects regression with repeated measures of the biomarker values (at baseline and 3 months) as the outcome variable. The main predictors included in this model will be randomization group, visit (baseline or 3 months) and the group*visit interaction. A subject-specific (random) intercept will be included to model heterogeneity in marker levels. A significant group×time interaction for an intervention will indicate that biomarker changes differ between the intervention and control groups. Additional covariates, that is, stratification variables (obesity status and employment status) and any factors found to be imbalanced between treatment groups at baseline will be included to examine the impact of covariates on estimated treatment effects. By using appropriate contrasts, intervention effect estimates (and 95% CIs) for the group comparisons of primary interest, that is, mean differences between each of the sitting interruption interventions with the control, will be calculated. To model multiple outcomes, we will use the sum of biomarker z-scores as a single outcome in the models, as described earlier, and explore O’Brien test [67] for multiple outcomes and multivariate mixed models. A similar analysis will be conducted for the secondary outcomes, namely, physical, emotional, cognitive functioning, and depressive symptoms, at 0 and 3 months.

For sensitivity analyses, we will include an indicator variable for COVID-19 (yes or no) and interactions, to test if participants who received altered protocols (eg, remote assessments) because of COVID-19 had similar changes compared with those who received the originally planned protocol.

For secondary analysis, we will include the targeted sedentary behavior (eg, minutes spent sitting, number of sit-to-stand transitions) as a time-varying covariate in the mixed models to test dose-response effects, that is, if a greater change in sedentary behavior is associated with greater change in biomarker or blood pressure outcomes.

##### Exploratory Moderator and Mediation Analysis

Moderators (eg, age) will be tested by including 3-way interaction terms between the putative moderator, time, and treatment condition in the mixed models described in the RCT analysis plan. In addition, accelerometer and activPAL days are nested within participants; therefore, when examining changes in sedentary behavior (outcome), we will further account for this hierarchical structure in the model. The device wear time will be entered as a fixed effect.

To assess whether psychosocial factors (eg, self-reported sleep) statistically mediate the effects of the interventions on sedentary behavior, we will apply the 4-step causal mediation framework to obtain direct and indirect effects [68]. Bootstrap resampling will be used to compute SEs and to test the significance of indirect or mediated effects [69].

##### Comparison of 2 Intervention Arms

By using appropriate contrasts in the RCT analysis mixed models, intervention effect estimates (and 95% CIs) for the 2 intervention conditions, that is, the mean differences in outcomes between the sit-to-stand and increased standing arms, will be calculated. The 95% CIs for these contrasts will be useful for quantifying the degree to which these interventions have equivalent effects on the biomarkers. For this analysis, we will conduct an intent-to-treat analysis and per-protocol analysis, as protocol violations and informative dropouts could bias the results toward equivalence.

## Results

Recruitment began in May 2018 and is currently ongoing. Data collection is expected to continue through 2022. Biomarker assays will be run thereafter, and data analysis and results are expected at the end of 2022.

## Discussion

### Principal Findings

Rise for Health will examine whether interrupting sitting time through 2 different behavioral changes in overweight postmenopausal women can impact glycemic control biomarkers of healthy aging and improve physical, emotional, and cognitive functioning. This is the first large-scale RCT to investigate the unique effects of brief sit-to-sand transitions in a real-world setting in postmenopausal women [25,70]. This study will provide important, new evidence to help inform public health and guide clinical and occupational health recommendations regarding the specific health effects of different ways of interrupting sitting.

Despite gaps in the evidence, public health agencies worldwide recommend reductions in sedentary behavior [71,72]. Many agencies suggest engaging in PA as a way to reduce sedentary time, although the goal of increasing exercise may not be possible for many older adults [73]. The World Health Organization recommends that adults aged 65 years or older limit the amount of time being sedentary and replace sedentary time with PA of any intensity and that older adults do more MVPA than recommended as a way to offset the negative impact of sedentary behavior [71]. However, there are many ways to reduce sedentary time, and it is unknown what types of alternate behaviors are beneficial to health and well-being. Disrupting sedentary time by sitting less or more sit-to-stand transitions may be more feasible behavior targets as they do not involve high-intensity or high-impact activity and may be more realistic for populations with some physical limitations. To date, most studies investigating sedentary time in older adults have been small, cross-sectional studies, with only a handful of intervention studies [25]. Little is known about the impact of interrupting sitting behaviors on emotional, cognitive, and functional outcomes, which are important for quality of life and healthy aging [74,75]. Although some previous studies have shown promising improvements on health outcomes, there has been a call for more rigorous study designs in large samples to study the effects of physical function, quality of life, disease risk, and healthy aging in older adults in a real-world, nonoccupational setting [25,70,76].

There is growing evidence of the importance of sit-to-stand transitions for health, distinct from sedentary time. Laboratory studies have shown that the frequency of disrupting sitting is important and that brief interruptions can increase postural blood flow and muscle contraction and induce changes in blood pressure, heart rate, and vascular tone [77,78]. Our pilot work findings support the paradigm that reducing sitting and increasing sit-to-stand transitions are 2 independent behaviors that require distinct and specific interventions [21]. Previous intervention studies have shown that a decrease in time spent sitting has no effect on sit-to-stand transitions [22,79]. These interventions have generally been aimed at decreasing sedentary time without focusing on sit-to-stand behavior and delivering mixed messages, encouraging participants to sit less, stand more, take breaks, and move more. This general approach makes it difficult to determine which types of alternative behaviors are linked to improved health. Therefore, it is important to study the specific behavioral targets of reducing sitting time and increasing brief transitions independently to examine how these different behaviors impact a variety of health outcomes.

The study limitations will be addressed whenever possible. Difficulty in recruiting a diverse sample or meeting our enrollment goal due to COVID-19 may limit the generalizability of the findings. This study is specifically enrolling older women; therefore, the results may not apply to men or younger women. Other limitations related to the COVID-19 pandemic are currently unknown but may occur if study protocols shift to support remote intervention delivery to maintain safety for participants and study staff. As the limited number of trials published to date are short in duration with small sample sizes, it is not known whether a 3-month intervention is long enough to support changes in our primary and secondary outcomes. A further limitation is that we are not assessing long-term effects or maintenance of effects in this study. A multilevel intervention using policy changes and environmental cues may improve long-term adherence and exceed the individual approach in this study.

### Conclusions

Rise for Health is a free-living intervention, RCT, within the STAR program grant, an National Institute of Aging funded program grant designed to provide more rigorous and comprehensive evidence on how to interrupt the sitting time and maximize positive impacts on healthy aging. Overall, this program grant aims to encourage a shift away from energy expenditure as the primary mechanism for health outcomes of reduced sitting to investigate sitting behaviors such as brief sit-to-stand transitions that expend little energy but engage muscles and improve postural blood flow and may impact physical functioning in older adults. Results from Rise for Health will be combined with results from our other STAR projects to collectively inform public health guidelines, occupational health practices, and related policies. By studying 2 distinct behaviors and a wide range of aging-related outcomes, we will be able to better understand intervention-specific differences in outcomes and, therefore, provide specific guidelines for which alternative behaviors impact what aspects of health and well-being.

## Appendix

Multimedia Appendix 1 Measurement and sample intervention schedule.

Multimedia Appendix 2 Peer-review report.

## Abbreviations

HOMA-IR: homeostatic model assessment of insulin resistance
MVPA: moderate-to-vigorous physical activity
NIH: National Institutes of Health
OPACH: Objective Physical Activity and Cardiovascular Disease Health in Older Women
PA: physical activity
PROMIS: Patient-Reported Outcomes Measurement Information System
RCT: randomized controlled trial
SPPB: Short Physical Performance Battery
STAR: Sedentary Time and Aging Mortality and Physical Function
UC San Diego: University California, San Diego

## References

[1] Matthews CE, Chen KY, Freedson PS, Buchowski MS, Beech BM, Pate RR, Troiano RP (2008). Amount of time spent in sedentary behaviors in the United States, 2003-2004. Am J Epidemiol.

[2] Hung WW, Ross JS, Boockvar KS, Siu AL (2011). Recent trends in chronic disease, impairment and disability among older adults in the United States. BMC Geriatr.

[3] Biswas A, Oh PI, Faulkner GE, Bajaj RR, Silver MA, Mitchell MS, Alter DA (2015). Sedentary time and its association with risk for disease incidence, mortality, and hospitalization in adults: a systematic review and meta-analysis. Ann Intern Med.

[4] Bankoski A, Harris TB, McClain JJ, Brychta RJ, Caserotti P, Chen KY, Berrigan D, Troiano RP, Koster A (2011). Sedentary activity associated with metabolic syndrome independent of physical activity. Diabetes Care.

[5] Carson V, Wong SL, Winkler E, Healy GN, Colley RC, Tremblay MS (2014). Patterns of sedentary time and cardiometabolic risk among Canadian adults. Prev Med.

[6] Copeland JL, Ashe MC, Biddle SJ, Brown WJ, Buman MP, Chastin S, Gardiner PA, Inoue S, Jefferis BJ, Oka K, Owen N, Sardinha LB, Skelton DA, Sugiyama T, Dogra S (2017). Sedentary time in older adults: a critical review of measurement, associations with health, and interventions. Br J Sports Med.

[7] Gao X, Nelson ME, Tucker KL (2007). Television viewing is associated with prevalence of metabolic syndrome in Hispanic elders. Diabetes Care.

[8] Healy GN, Matthews CE, Dunstan DW, Winkler EA, Owen N (2011). Sedentary time and cardio-metabolic biomarkers in US adults: NHANES 2003-06. Eur Heart J.

[9] Keadle SK, Conroy DE, Buman MP, Dunstan DW, Matthews CE (2017). Targeting reductions in sitting time to increase physical activity and improve health. Med Sci Sports Exerc.

[10] Wirth K, Klenk J, Brefka S, Dallmeier D, Faehling K, Figuls MR, Tully MA, Giné-Garriga M, Caserotti P, Salvà A, Rothenbacher D, Denkinger M, Stubbs B, SITLESS consortium (2017). Biomarkers associated with sedentary behaviour in older adults: a systematic review. Ageing Res Rev.

[11] Chang YJ, Bellettiere J, Godbole S, Keshavarz S, Maestas JP, Unkart JT, Ervin D, Allison MA, Rock CL, Patterson RE, Jankowska MM, Kerr J, Natarajan L, Sears DD (2020). Total sitting time and sitting pattern in postmenopausal women differ by hispanic ethnicity and are associated with cardiometabolic risk biomarkers. J Am Heart Assoc.

[12] Rice DP (2000). Older women's health and access to care. Womens Health Issues.

[13] Nelson ME, Rejeski WJ, Blair SN, Duncan PW, Judge JO, King AC, Macera CA, Castaneda-Sceppa C, American College of Sports Medicine, American Heart Association (2007). Physical activity and public health in older adults: recommendation from the American College of Sports Medicine and the American Heart Association. Circulation.

[14] Troiano RP, Berrigan D, Dodd KW, Mâsse Louise C, Tilert T, McDowell M (2008). Physical activity in the United States measured by accelerometer. Med Sci Sports Exerc.

[15] Dunlop DD, Song J, Arnston EK, Semanik PA, Lee J, Chang RW, Hootman JM (2015). Sedentary time in US older adults associated with disability in activities of daily living independent of physical activity. J Phys Act Health.

[16] Pahor M, Guralnik JM, Ambrosius WT, Blair S, Bonds DE, Church TS, Espeland MA, Fielding RA, Gill TM, Groessl EJ, King AC, Kritchevsky SB, Manini TM, McDermott MM, Miller ME, Newman AB, Rejeski WJ, Sink KM, Williamson JD, LIFE study investigators (2014). Effect of structured physical activity on prevention of major mobility disability in older adults: the LIFE study randomized clinical trial. J Am Med Assoc.

[17] Brawley LR, Rejeski WJ, King AC (2003). Promoting physical activity for older adults: the challenges for changing behavior. Am J Prev Med.

[18] Ashe MC, Winters M, Hoppmann CA, Dawes MG, Gardiner PA, Giangregorio LM, Madden KM, McAllister MM, Wong G, Puyat JH, Singer J, Sims-Gould J, McKay HA (2015). "Not just another walking program": Everyday Activity Supports You (EASY) model-a randomized pilot study for a parallel randomized controlled trial. Pilot Feasibility Stud.

[19] Brierley ML, Chater AM, Smith LR, Bailey DP (2019). The effectiveness of sedentary behaviour reduction workplace interventions on cardiometabolic risk markers: a systematic review. Sports Med.

[20] Rosenberg DE, Gell NM, Jones SM, Renz A, Kerr J, Gardiner PA, Arterburn D (2015). The feasibility of reducing sitting time in overweight and obese older adults. Health Educ Behav.

[21] Kerr J, Takemoto M, Bolling K, Atkin A, Carlson J, Rosenberg D, Crist K, Godbole S, Lewars B, Pena C, Merchant G (2016). Two-arm randomized pilot intervention trial to decrease sitting time and increase sit-to-stand transitions in working and non-working older adults. PLoS One.

[22] Fitzsimons CF, Kirk A, Baker G, Michie F, Kane C, Mutrie N (2013). Using an individualised consultation and activPAL™ feedback to reduce sedentary time in older Scottish adults: results of a feasibility and pilot study. Prev Med.

[23] Gardiner PA, Eakin EG, Healy GN, Owen N (2011). Feasibility of reducing older adults' sedentary time. Am J Prev Med.

[24] Lewis LK, Rowlands AV, Gardiner PA, Standage M, English C, Olds T (2016). Small Steps: preliminary effectiveness and feasibility of an incremental goal-setting intervention to reduce sitting time in older adults. Maturitas.

[25] Aunger JA, Doody P, Greig CA (2018). Interventions targeting sedentary behavior in non-working older adults: a systematic review. Maturitas.

[26] Harrington DM, Barreira TV, Staiano AE, Katzmarzyk PT (2014). The descriptive epidemiology of sitting among US adults, NHANES 2009/2010. J Sci Med Sport.

[27] Owen N, Healy GN, Matthews CE, Dunstan DW (2010). Too much sitting: the population health science of sedentary behavior. Exerc Sport Sci Rev.

[28] Thorp AA, Owen N, Neuhaus M, Dunstan DW (2011). Sedentary behaviors and subsequent health outcomes in adults a systematic review of longitudinal studies, 1996-2011. Am J Prev Med.

[29] Hartman SJ, Marinac CR, Cadmus-Bertram L, Kerr J, Natarajan L, Godbole S, Patterson RE, Morey B, Sears DD (2018). Sedentary behaviors and biomarkers among breast cancer survivors. J Phys Act Health.

[30] Harris PA, Scott KW, Lebo L, Hassan N, Lightner C, Pulley J (2012). ResearchMatch: a national registry to recruit volunteers for clinical research. Acad Med.

[31] Gardner B, Phillips LA, Judah G (2016). Habitual instigation and habitual execution: definition, measurement, and effects on behaviour frequency. Br J Health Psychol.

[32] Gardner B, Smith L, Aggio D, Iliffe S, Fox KR, Jefferis BJ, Hamer M (2015). 'On Your Feet to Earn Your Seat': update to randomised controlled trial protocol. Trials.

[33] Phillips LA, Gardner B (2016). Habitual exercise instigation (vs. execution) predicts healthy adults' exercise frequency. Health Psychol.

[34] Matei R, Thuné-Boyle I, Hamer M, Iliffe S, Fox KR, Jefferis BJ, Gardner B (2015). Acceptability of a theory-based sedentary behaviour reduction intervention for older adults ('On Your Feet to Earn Your Seat'). BMC Public Health.

[35] Bandura A (1986). Social Foundations of Thought and Action: A Social Cognitive Theory.

[36] Remote administration guidelines for the NIH Toolbox®: response to COVID-19.

[37] Obeid JS, McGraw CA, Minor BL, Conde JG, Pawluk R, Lin M, Wang J, Banks SR, Hemphill SA, Taylor R, Harris PA (2013). Procurement of shared data instruments for Research Electronic Data Capture (REDCap). J Biomed Inform.

[38] Harris PA, Taylor R, Thielke R, Payne J, Gonzalez N, Conde JG (2009). Research electronic data capture (REDCap)--a metadata-driven methodology and workflow process for providing translational research informatics support. J Biomed Inform.

[39] Harris PA, Taylor R, Minor BL, Elliott V, Fernandez M, O'Neal L, McLeod L, Delacqua G, Delacqua F, Kirby J, Duda SN, REDCap Consortium (2019). The REDCap consortium: building an international community of software platform partners. J Biomed Inform.

[40] Lyden K, Keadle SL, Staudenmayer JW, Freedson PS (2012). Validity of two wearable monitors to estimate breaks from sedentary time. Med Sci Sports Exerc.

[41] Kim Y, Barry VW, Kang M (2015). Validation of the ActiGraph GT3X and activPAL accelerometers for the assessment of sedentary behavior. Meas Phys Educ Exerc Sci.

[42] Grant PM, Ryan CG, Tigbe WW, Granat MH (2006). The validation of a novel activity monitor in the measurement of posture and motion during everyday activities. Br J Sports Med.

[43] Kozey-Keadle S, Libertine A, Lyden K, Staudenmayer J, Freedson PS (2011). Validation of wearable monitors for assessing sedentary behavior. Med Sci Sports Exerc.

[44] Janz KF (1994). Validation of the CSA accelerometer for assessing children's physical activity. Med Sci Sports Exerc.

[45] Melanson EL, Freedson PS (1995). Validity of the Computer Science and Applications, Inc. (CSA) activity monitor. Med Sci Sports Exerc.

[46] Plasqui G, Westerterp KR (2007). Physical activity assessment with accelerometers: an evaluation against doubly labeled water. Obesity (Silver Spring).

[47] Choi L, Liu Z, Matthews CE, Buchowski MS (2011). Validation of accelerometer wear and nonwear time classification algorithm. Med Sci Sports Exerc.

[48] Evenson KR, Wen F, Herring AH, Di C, LaMonte MJ, Tinker LF, Lee I, Rillamas-Sun E, LaCroix AZ, Buchner DM (2015). Calibrating physical activity intensity for hip-worn accelerometry in women age 60 to 91 years: the Women's Health Initiative OPACH Calibration Study. Prev Med Rep.

[49] Guralnik JM, Simonsick EM, Ferrucci L, Glynn RJ, Berkman LF, Blazer DG, Scherr PA, Wallace RB (1994). A short physical performance battery assessing lower extremity function: association with self-reported disability and prediction of mortality and nursing home admission. J Gerontol.

[50] Katz S, Downs TD, Cash HR, Grotz RC (1970). Progress in development of the index of ADL. Gerontologist.

[51] Katz S (1983). Assessing self-maintenance: activities of daily living, mobility, and instrumental activities of daily living. J Am Geriatr Soc.

[52] Ware JE, Sherbourne CD (1992). The MOS 36-item short-form health survey (SF-36). I. Conceptual framework and item selection. Med Care.

[53] Radloff LS (2016). The CES-D scale. Appl Psychol Meas.

[54] Heaton RK, Akshoomoff N, Tulsky D, Mungas D, Weintraub S, Dikmen S, Beaumont J, Casaletto KB, Conway K, Slotkin J, Gershon R (2014). Reliability and validity of composite scores from the NIH Toolbox Cognition Battery in adults. J Int Neuropsychol Soc.

[55] Weintraub S, Dikmen SS, Heaton RK, Tulsky DS, Zelazo PD, Slotkin J, Carlozzi NE, Bauer PJ, Wallner-Allen K, Fox N, Havlik R, Beaumont JL, Mungas D, Manly JJ, Moy C, Conway K, Edwards E, Nowinski CJ, Gershon R (2014). The cognition battery of the NIH toolbox for assessment of neurological and behavioral function: validation in an adult sample. J Int Neuropsychol Soc.

[56] Lai JS, Wagner LI, Jacobsen PB, Cella D (2014). Self-reported cognitive concerns and abilities: two sides of one coin?. Psychooncology.

[57] Yu L, Buysse DJ, Germain A, Moul DE, Stover A, Dodds NE, Johnston KL, Pilkonis PA (2011). Development of short forms from the PROMIS™ sleep disturbance and Sleep-Related Impairment item banks. Behav Sleep Med.

[58] Sechrist KR, Walker SN, Pender NJ (1987). Development and psychometric evaluation of the exercise benefits/barriers scale. Res Nurs Health.

[59] Sallis JF, Pinski RB, Grossman RM, Patterson TL, Nader PR (1988). The development of self-efficacy scales for healthrelated diet and exercise behaviors. Health Educ Res.

[60] Stewart AL, Mills KM, King AC, Haskell WL, Gillis D, Ritter PL (2001). CHAMPS physical activity questionnaire for older adults: outcomes for interventions. Med Sci Sports Exerc.

[61] Pilkonis PA, Choi SW, Reise SP, Stover AM, Riley WT, Cella D, PROMIS Cooperative Group (2011). Item banks for measuring emotional distress from the Patient-Reported Outcomes Measurement Information System (PROMIS®): depression, anxiety, and anger. Assess.

[62] Feldman G, Hayes A, Kumar S, Greeson J, Laurenceau J (2006). Mindfulness and emotion regulation: the development and initial validation of the Cognitive and Affective Mindfulness Scale-Revised (CAMS-R). J Psychopathol Behav Assess.

[63] Amtmann D, Cook KF, Jensen MP, Chen W, Choi S, Revicki D, Cella D, Rothrock N, Keefe F, Callahan L, Lai J (2010). Development of a PROMIS item bank to measure pain interference. Pain.

[64] Chen W, Revicki D, Amtmann D, Jensen M, Keefe F, Cella D (2012). Development and analysis of PROMIS pain intensity scale. Qual Life Res.

[65] Gardner B, Abraham C, Lally P, de Bruijn G (2012). Towards parsimony in habit measurement: testing the convergent and predictive validity of an automaticity subscale of the Self-Report Habit Index. Int J Behav Nutr Phys Act.

[66] Verplanken B, Orbell S (2006). Reflections on past behavior: a self-report index of habit strength. J Appl Soc Psychol.

[67] O'Brien PC (1984). Procedures for comparing samples with multiple endpoints. Biometrics.

[68] Bind MC, Vanderweele TJ, Coull BA, Schwartz JD (2016). Causal mediation analysis for longitudinal data with exogenous exposure. Biostatistics.

[69] Efron B, Tibshirani RJ (1994). An Introduction to the Bootstrap.

[70] Taylor W, Rix K, Gibson A, Paxton R (2020). Sedentary behavior and health outcomes in older adults: a systematic review. AIMS Medical Science.

[71] World Health Organization (2020). WHO Guidelines on Physical Activity and Sedentary Behaviour.

[72] Brown WJ, Bauman AE, Bull FC, Burton NW (2012). Development of evidence-based physical activity recommendations for adults (18-64 years). Report prepared for the Australian Government Department of Health.

[73] Prince SA, Saunders TJ, Gresty K, Reid RD (2014). A comparison of the effectiveness of physical activity and sedentary behaviour interventions in reducing sedentary time in adults: a systematic review and meta-analysis of controlled trials. Obes Rev.

[74] Vance DE, Wadley VG, Ball KK, Roenker DL, Rizzo M (2005). The effects of physical activity and sedentary behavior on cognitive health in older adults. J Aging Phys Act.

[75] Voss MW, Carr LJ, Clark R, Weng T (2014). Revenge of the “sit” II: does lifestyle impact neuronal and cognitive health through distinct mechanisms associated with sedentary behavior and physical activity?. Ment Health Phy Act.

[76] Shrestha N, Grgic J, Wiesner G, Parker A, Podnar H, Bennie JA, Biddle SJ, Pedisic Z (2019). Effectiveness of interventions for reducing non-occupational sedentary behaviour in adults and older adults: a systematic review and meta-analysis. Br J Sports Med.

[77] Olufsen MS, Ottesen JT, Tran HT, Ellwein LM, Lipsitz LA, Novak V (2005). Blood pressure and blood flow variation during postural change from sitting to standing: model development and validation. J Appl Physiol (1985).

[78] Castellano V, Olive JL, Stoner L, Black C, McCully KK (2004). Blood flow response to a postural challenge in older men and women. Dyn Med.

[79] Healy GN, Eakin EG, Lamontagne AD, Owen N, Winkler EA, Wiesner G, Gunning L, Neuhaus M, Lawler S, Fjeldsoe BS, Dunstan DW (2013). Reducing sitting time in office workers: short-term efficacy of a multicomponent intervention. Prev Med.

