# Supplementary figures and images for: Interrupting Sitting Time in Postmenopausal Women: Protocol for the Rise for Health Randomized Controlled Trial
Source: JMIR Res Protoc. 2021 May 13;10(5):e28684. doi: 10.2196/28684 (PMC8160808; doi:10.2196/28684)

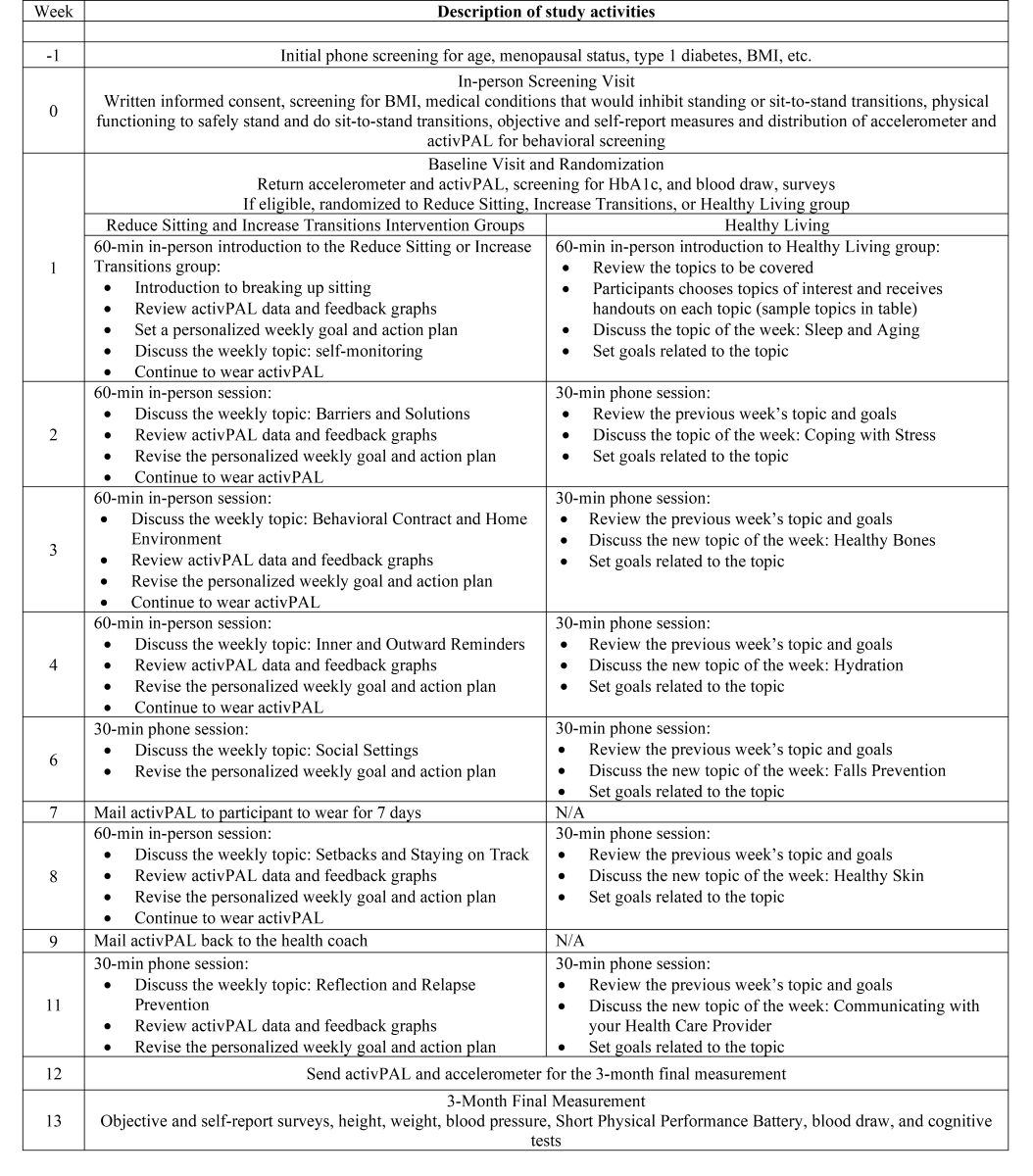

Supplement: Multimedia Appendix 1 [file resprot_v10i5e28684_app1.png]
