# Supplementary material for: Interrupting Sitting Time in Postmenopausal Women: Protocol for the Rise for Health Randomized Controlled Trial
Source: JMIR Res Protoc. 2021 May 13;10(5):e28684. doi: 10.2196/28684 (PMC8160808; doi:10.2196/28684)
Supplement: Multimedia Appendix 2 [file resprot_v10i5e28684_app2.pdf]

SUMMARY STATEMENT
( Privileged Communication )

PROGRAM CONTACT:
SERGEI ROMASHKAN
(301) 435-3047
romashks@nia.nih.gov

*Release Date:* 12/09/2016
*Revised Date:*

*Application Number:* 1 P01 AG052352-01A1

Principal Investigators (Listed Alphabetically):

KERR, JACQUELINE
LACROIX, ANDREA Z. (Contact)

Applicant Organization: UNIVERSITY OF CALIFORNIA SAN DIEGO

*Review Group:* ZAG1 ZIJ-7 (J1)
National Institute on Aging Special Emphasis Panel
Second Stage P01 Review

*Meeting Date:* 11/21/2016
*Council:* JAN 2017
*Requested Start:* 04/01/2017
*RFA/PA:* PAR13-258
*PCC:* 4CCTSSR

*Project Title:* Sedentary Behaviour Interrupted: Acute, medium and long-term effects on biomarkers of healthy aging, physical function and mortality
*SRG Action:* Impact Score:20
*Next Steps:* Visit http://grants.nih.gov/grants/next_steps.htm
Human Subjects: 30-Human subjects involved - Certified, no SRG concerns
Animal Subjects: 10-No live vertebrate animals involved for competing appl.
Gender: 2A-Only women, scientifically acceptable
Minority: 1A-Minorities and non-minorities, scientifically acceptable
Children: 3A-No children included, scientifically acceptable
Clinical Research - not NIH-defined Phase III Trial

| Project Year | Direct Costs Requested | Estimated Total Cost |
|---|---|---|
| 1 | 1,499,411 | 2,328,592 |
| 2 | 1,499,350 | 2,328,498 |
| 3 | 1,499,882 | 2,329,324 |
| 4 | 1,499,826 | 2,329,237 |
| 5 | 1,499,645 | 2,328,956 |
| TOTAL | 7,498,114 | 11,644,606 |

ADMINISTRATIVE BUDGET NOTE: The budget shown is the requested budget and has not been adjusted to reflect any recommendations made by reviewers. If an award is planned, the costs will be calculated by Institute grants management staff based on the recommendations outlined below in the COMMITTEE BUDGET RECOMMENDATIONS section.

**PROJECT 2: SEDENTARY BEHAVIOR INTERRUPTED: A RANDOMIZED TRIAL OF 3-MONTH EFFECTS OF BIOMARKERS OF HEALTHY AGING AND PHYSICAL FUNCTIONING IN THE REAL WORLD; Dr. Jacqueline Kerr, Project Leader (PL)**

**DESCRIPTION (provided by applicant):** Project 2 of the Sedentary Time and Aging Research (STAR) Program will investigate how 3 month changes in standing time, brief sit-to-stand transitions and physical activity (PA) breaks, will impact biomarkers of healthy aging, and physical, emotional and cognitive functioning compared to healthy living attention controls. This 4-arm randomized controlled trial (RCT) will occur in the `real world' with postmenopausal women (N=592) who are not physically active, do not stand up frequently and spend at least 8 hours sitting per day, i.e. most older adults. The daily behavior targets in each condition of Project 2 (increase standing by 2 hours, increase sit-to-stand transitions by 30 per day, & increase PA breaks by 12) reflect the treatment conditions explored in Project 1. To date, there have been no RCT studies with health outcomes in older adults. Our multiple pilot studies, however, demonstrate the feasibility and acceptability of delivering intervention materials (including standing desks, trackers & ActivPAL feedback) to older adults and evaluating behavior change objectively with thigh-worn inclinometers. The interventions significantly reduced sitting time (up to 2 hours a day) and significantly increased sit-to-stand transitions (up to 40 per day). Increases in PA were minimal in the standing conditions, compared to our PA focused RCT in 307 older adults which increased short PA bouts. One pre-post pilot found that reductions in sitting time were significantly related to improved gait speed and decreased depressive symptoms. Project 2 will assess the impact of the 3 interventions to interrupt sitting compared to the attention control condition at 6 and 12 weeks. We will recruit 592 overweight, sedentary, postmenopausal women who will be randomized to one of the 4 conditions. We will employ tools such as standing desks, wrist-worn device alerts and condition-specific, tailored ActivPAL feedback. The primary outcomes are glucose regulation and blood pressure. Secondary outcomes include mitochondrial functioning (measured by muscle mitochondrial respiratory capacity), and physical, emotional and cognitive functioning. Exploratory outcomes include psychosocial and environmental mediators and moderators of behavior change. We will also explore the moderating effect of age on the intervention outcomes. Project 2 compliments the other projects by assessing 3 month intermediary outcomes. Project 2 will help validate the novel computational algorithms optimized in Project 1 and applied to Project 3. In addition, the Biostatistics Core will investigate the response patterns in Projects 1 and 3 and map them to Project 2. Project 2 will strengthen the public health evidence of how to interrupt sitting time in older adults, provide specific information on interrupting sitting as an alternative to longer PA bouts, and explore a range of targeted outcomes related to healthy aging.

**CRITIQUE 1:**

Significance: 2
Investigator(s): 1
Innovation: 1
Approach: 4
Environment: 1

**Overall Impact:**
This updated and revised Project 2 application aims to compare three interventions (increased standing, increased sit-to-stand transitions, and increased PA bouts) to disrupt sitting with a control group on markers of glucose regulation and blood pressure. As secondary aims, the investigators will assess whether these interventions impact muscle mitochondrial capacity; physical, emotional and cognitive outcomes. Finally, the effect of age, psychosocial and environmental factors on these relationships will be examined and the outcomes will be compared between interventions. This is an application from an experienced investigative team with a successful record of publications and funding

in the area of physical activity and sedentary behavior. The PL, Co-Is and consultants are all well positioned to successfully complete this project and disseminate the results. The research question within this application has important clinical, scientific and practical significance. The investigators were responsive to the previous review. However, changes to the physical activity intervention and a lack of detailed descriptions of the interventions has diminished the reviewer's enthusiasm for this application.

**1. Significance:**
**Strengths**
- Interventions that focus on interrupting or decreasing time spent in sedentary behaviors in this population may show a clinical benefit.
- The population of focus in this application would likely benefit greatly from reductions in sitting and increases in movement.
- Comparing changes in behaviors and biomarkers in response to interventions that aim to reduce sitting, increase disruptions to sedentary behavior, and increase physical activity will be highly informative to future interventions.
- Few studies have examined the impact of disrupting sitting time outside of the laboratory, therefore the results of this project will provide information on the efficacy of these interventions as well as on the outcomes listed.

**Weaknesses**
- The PA intervention changed from increasing PA by 30 min/day to taking 12 extra 2-5 min PA breaks per day. This is still a break to (potentially) sedentary behavior and may not be distinct enough from the other intervention conditions.

**2. Investigator(s):**
**Strengths**
- The PL is a mid-career investigator with a strong publication and funding history in this area of inquiry.
- The team has successfully worked together in the past and continue to work together on other projects.
- There appears to be sufficient and qualified personnel to carry out the research project (Health Educator, Project Coordinator, Recruiter, etc.).

**Weaknesses**
- None noted.

**3. Innovation:**
**Strengths**
- This Project will be the first RCT to examine and compare the effects of three interventions to disrupt and/or reduce sedentary behavior in post-menopausal women.
- There is a lack of RCTs examining interruptions to sitting.
- This project will be the first to examine the impact of using standing desks outside of the workplace or school to reduce sitting behavior.
- This project will compare three interventions to disrupt sitting- one with standing, one with increased sit-to-stand transitions, and one with physical activity.
- Comparing the effect of these interventions on muscle mitochondrial function is novel.

**Weaknesses**
- None noted.

**4. Approach:**
**Strengths**
- The use of valid and reliable measures of SB and PA is a strength.

- The application of RCT is a strong design, with a strong rationale supporting separation of standing and sit-to-stand transitions as separate interventions. This design and the outcome of this study will give researchers and clinicians an indication the effectiveness of sitting interruption interventions. Further, comparing 2 sedentary interventions to a PA group and a control group may allow the investigators to answer many questions about the efficacy of the standing interventions vs. PA interventions, pending distinctness of the interventions.
- The researchers have tried to isolate each intervention behavior – standing, transitions from sitting to standing, and PA.
- The use of the CTRI, the new AHA Center, and the numerous other Center connections will strengthen the application.
- The control group intervention is strong, with a clear indication of information that will be delivered.
- The inclusion/exclusion criteria pertaining to sitting, sit-to-stand transitions and physical activity are strong and will result in a study population that is highly likely to benefit from the intervention.
- The screening process is well described and thorough.

**Weaknesses**

- The descriptions of the interventions are lacking. It is not clear what will be asked of the participants in the intervention arms. Are the participants given suggestions on what to do to once they stand up? Will they be restricted to standing only? What types of PA will be recommended? Further, is it not clear how distinct the interventions will be from each other, once the participants start carrying out the interventions. The potential overlap in the interventions will result in an inability to decipher the unique effects on the outcomes of interest.
- The rationale for the 2-5 min PA breaks is not clear. This PA recommendation is not in line with the PA recommendations.
- The dose of activity in each of these interventions is not equated, which will make it difficult to compare the outcome to standing, sit-to-stand transitions, to PA.
- The validation of machine learning algorithms is not well integrated into the application.

**5. Environment:**

**Strengths**

- The University, the CTRI, and the Centers are well- equipped and supportive of the successful completion of this application.
- Outstanding.

**Weaknesses**

- None noted.

**Protections for Human Subjects:**

Acceptable risks and adequate protections.

**Data and Safety Monitoring Plan:**

Acceptable.

- While a DSMP is not needed, it provides and additional layer of protection for the research participants.

**Inclusion of Women, Minorities and Children:**

- Sex/Gender: Distribution justified scientifically.
- Race/Ethnicity: Distribution justified scientifically.
- For NIH-Defined Phase III trials, Plans for valid design and analysis: Not applicable.
- Inclusion/Exclusion of Children under 18: Excluding ages <18; justified scientifically.

- The inclusion of women only is justified in the application.

**Resubmission:**

- The investigators address most of the comments provided by the reviewers. However, one arm of the intervention was changed from a 30-minute PA intervention to 12, 2-5 minute PA breaks. The reviewer is not convinced this will be distinct enough from the sit-to-stand intervention.
- The interventions are not well described. It is not clear what the participants will be told to do in each of the interventions. For instance, if they stand up, can they move to go get a drink of water?
- With the removal of the biomarkers core, there were 4 additional personnel added to this Project, which will strengthen the quality and dissemination of the project.

**Budget and Period of Support:**
Recommend as Requested.

**CRITIQUE 2:**

Significance: 1
Investigator(s): 1
Innovation: 2
Approach: 5
Environment: 1

**1. Significance**:
**Strengths**

- Sedentary behavior is important risk factor for older adults and there is a need to understand lifestyle approaches to avert these effects.
- Results have the potential to inform lifestyle approaches to reduce negative health effects of sedentary behavior.

**Weakness**

- None noted.

**2. Investigators**:
**Strengths**

- The investigative team is outstanding and well qualified to conduct the proposed trial.

**Weakness**

- None noted.

**3. Innovation:**
**Strengths**

- This will be the first RCT to focus on changing sedentary behavior in overweight postmenopausal women.
- Multiple approaches will be assessed.
- Concurrent measures of glycemic control and endothelial function outcomes.

**Weaknesses**

- Although post-menopausal women have not been studied before in a randomized trial, it is not abundantly clear why other older adults at risk are not included.

**4. Approach:**
**Strengths**

- Use of mobile tools to track sitting interruptions in real time and also summarized at the daily/weekly level.
- Feasibility supported by large-scale pilot trials.

**Weaknesses**

- The rationale for the PA condition is confusing. It is stated that the 2-5 min breaks were selected to be in line with the PA guidelines. However, the PA guidelines are focused on accumulation of moderate intensity activity in bouts of 10 min or more. Thus, the investigators related to the accumulation of moderate to vigorous intensity activity of 30 min over the course of a day. However, the bouts are referred to a "light" PA bouts which would not be consistent or related to PA guidelines. The intensity of the PA breaks is not described. If light intensity, then the recommended PA breaks are not consistent with current PA guidelines.
- Given that the total amount of time that participants take break from sedentary behavior vary across conditions, the findings of the proposed study may be difficult to interpret since it would not be clear if changes were due to a dose effect or type of activity effect.
- Power is limited to detect differences across intervention conditions. This is a significant weakness given the potential importance of this question for health recommendations for older adults.

**5. Environment**:

**Strengths:**

- The Environment to conduct the planned study is outstanding.

**Weaknesses**

- None noted.

**Protections for Human Subjects:**

Acceptable Risks and/or Adequate Protections.

- No concerns.

**Data and Safety Monitoring Plan:**

- Acceptable.

**Inclusion of Women, Minorities and Children:**

- Sex/Gender: Distribution justified scientifically.
- Race/Ethnicity: Distribution justified scientifically.
- For NIH-Defined Phase III trials, Plans for valid design and analysis: Not applicable.
- Inclusion/Exclusion of Children under 18: Excluding ages <18; justified scientifically.

**Vertebrate Animals:**

Not Applicable (No Vertebrate Animals).

**Resubmission:**

- Investigators were very responsive to recommendations from previous submission.

**Budget and Period of Support:**

Recommend as Requested.

**CRITIQUE 3:**

Significance: 2
Investigator(s): 1
Innovation: 1

Approach: 2
Environment: 1

**Overall Impact:**
The outcomes of this Project, if achieved, have important clinical and practical significance to the field of health promotion and PA. This is a well-written application from experienced team who have demonstrated by previous work that they are likely to achieve this ambitious project. Project 2 is innovative, comparing 3 interventions to attention/education controls. This Project will have multiple outcomes, from those demonstrating mechanisms of improvement in health from objective measures (NIRS, glucose regulation, BP) to self-reported physical, psychosocial, cognitive, and other exploratory outcomes. Enthusiasm for this project is high and is likely to have a substantial impact on the science of SB and PA and how they may improve health. Weaknesses are focused primarily on the approach. Main concerns include attrition of subjects, overlap of the conditions of the 3 arms, compliance with the intervention conditions by subjects.

**1. Significance:**
**Strengths**
- Developing effective interventions for older women, other than PA, that will improve health (glucose regulation, BP, etc.) is important to improve methods for lifestyle counseling.
- There is a need to study older adults who frequently have metabolic impairments and who could likely show maximal benefit from interrupting SB especially since many older adults do not regularly exercise due to perceived or real barriers.
- Improves scientific evidence of methods of interrupting SB and which method would be superior in terms of important metabolic outcomes.
- If this Project is successful and outcomes achieved, it is likely to have great impact on the field of health promotion & disease prevention.
- Improved rational for scientific evidence of outcomes

**Weaknesses**
- Not provided.

**2. Investigator(s):**
**Strengths**
- Successful team, demonstrated outcomes, highly qualified to conduct this ambitious program.
- Preliminary data and pilot studies performed provide confidence in ability to complete ambitious project.

**Weaknesses**
- None noted.

**3. Innovation:**
**Strengths**
- Comparing 3 treatment conditions of reducing SB and comparing metabolic outcomes of each group is highly innovative and not accomplished to date.
- Adding mitochondrial measures -NIRS- helps determine mechanisms of action of reducing SB and is innovative.
- Whether emotional and functional outcomes will improve by interrupting SB using different methods is an innovative question, yet particularly salient for older adults.

**Weaknesses**
- Interventions methods/components of changing SB behaviors not highly innovative and may not be pragmatic.

**4. Approach:**

**Strengths**

- 4 arm comparisons.
- Objective measures and self-reported measures for primary, secondary and exploring mediators and moderators.
- Shorter outcome time in resubmission to test interventions will lesson attrition and improve probability of success.
- Measures of adherence for each condition, important to measure satisfaction with protocol to inform future studies.
- Improved clarity of inclusion/exclusion criteria (and rationale for), measures of PA/SB with both ActivPAL and Actigraph.
- Investigators very responsive to suggestions from previous submission & improved methods.
- Biostatistics support excellent.

**Weaknesses**

- Intervention components are very ambitious. Not sure if older women will accept the burden of intervention components (many visits to medical center, multiple phone calls, blood draws; packaged dinners, nothing by mouth status); not sure they can accomplish the requirements of each arm, thus limiting variability in outcomes.
- Remained concerned with recruitment and retention of participants and 10% attrition rate is over ambitious (most PA trials at least~20%).
- There is a concern about experimental condition overlap; e.g. standing breaks and brief sit to stand transitions are very similar and subjects may not perform correctly, e.g. PA short breaks may be taken instead of just standing in place.
- Intervention not pragmatic, difficult to enact & would be difficult to replicate in clinical practice because each intervention is testing multiple components that have a menu of choices/different components for each woman to perform. Will each arm be representing the same treatment?
- 20 minute MVPA arm replaced with PA breaks; rational for the latter is lacking, more important to include one arm with the current recommendation (20 min MVPA).
- Motivational interviewing is mentioned as a behavioral strategy in intervention, but components not supported by Table 1, maybe just motivational components and self-efficacy.

**5. Environment:**
**Strengths**

- Superior research environment.

**Weaknesses**

- None noted.

**Protections for Human Subjects:**
Acceptable Risks and/or Adequate Protections.

**Data and Safety Monitoring Plan:**
Acceptable.

**Inclusion of Women, Minorities and Children:**

- Sex/Gender: Distribution justified scientifically.
- Race/Ethnicity: Distribution justified scientifically.
- For NIH-Defined Phase III trials, Plans for valid design and analysis: Not applicable.
- Inclusion/Exclusion of Children under 18: Excluding ages <18; justified scientifically.

**Vertebrate Animals:**
Not Applicable (No Vertebrate Animals).

**PROGRAM PROJECT AGGREGATE ROSTER**
**NATIONAL INSTITUTE ON AGING**
**January 2017 Council Round**

THE ROSTER INCLUDES MEMBERS FROM MULTIPLE NIA P01 SPECIAL EMPHASIS PANELS OF THIS IRG:

**CHAIRPERSON(S)**

ANDERSON, ROZALYN M., PHD
ASSOCIATE PROFESSOR
DEPARTMENT OF MEDICINE
DIVISION OF GERIATRICS
UNIVERSITY OF WISCONSIN
MADISON, WI 53706

ASTHANA, SANJAY, MD
PROFESSOR AND HEAD
DEPARTMENT OF GERIATRICS AND GERONTOLOGY
UNIVERSITY OF WISCONSIN SCHOOL OF MEDICINE
MADISON, WI 53705

COX, DONALD C., PHD
PROFESSOR
DEPARTMENT OF ECONOMICS
BOSTON COLLEGE
CHESTNUT HILL, MA 02467

CRIBBS, DAVID HASTINGS, PHD
PROFESSOR AND ASSOCIATE DIRECTOR
DEPARTMENT OF NEUROLOGY
THE INSTITUTE FOR MEMORY IMPAIRMENTS AND NEUROLOGICAL DISORDERS
UNIVERSITY OF CALIFORNIA, IRVINE
IRVINE, CA 92697

DEKOSKY, STEVEN T., MD
PROFESSOR OF NEUROLOGY
INTEREIM EXECUTIVE DIRECTOR
MCKNIGHT BRAIN INSTITUTE
UNIVERSITY OF FLORIDA
GAINESVILLE, FL 32610

DOW, WILLIAM HATFIELD, PHD
HENRY J. KAISER PROFESSOR AND DIRECTOR
CENTER ON THE ECONOMICS AND DEMOGRAPHY OF AGING
SCHOOL OF PUBLIC HEALTH
UNIVERSITY OF CALIFORNIA, BERKELEY
BERKELEY, CA 94720

HO, VIVIAN, PHD
PROFESSOR
DEPARTMENT OF ECONOMICS
JAMES A. BAKER III INSTITUTE CHAIR IN HEALTH ECONOMICS
INSTITUTE FOR PUBLIC POLICY
RICE UNIVERSITY
HOUSTON, TX 77251

KOHL, HAROLD WILLIS, PHD
PROFESSOR
DEPARTMENT OF KINESIOLOGY AND HEALTH EDUCATION
UNIVERSITY OF TEXAS AT AUSTIN
AUSTIN, TX 78701

LANE, JULIA INGRID, PHD
SENIOR MANAGING ECONOMIST
AMERICAN INSTITUTES FOR RESEARCH
WASHINGTON, DC 20007

LOESER, RICHARD F., MD
PROFESSOR
DIVISION OF RHEUMATOLOGY
THURSTON ARTHRITIS RESEARCH CENTER
UNIVERSITY OF NORTH CAROLINA
CHAPEL HILL, NC 27599

RECANZONE, GREGG HOWARD, PHD

PROFESSOR
SECTION OF NEUROBIOLOGY, PHYSIOLOGY AND BEHAVIOR CENTER FOR NEUROSCIENCE
UNIVERSITY OF CALIFORNIA, DAVIS
DAVIS, CA 95618

ROBBINS, PAUL D., PHD
PROFESSOR
DEPARTMENT OF METABOLISM AND AGING
THE SCRIPPS RESEARCH INSTITUTE
JUPITER, FL 33458

TATAR, MARC, PHD
PROFESSOR
DEPARTMENT OF ECOLOGY AND EVOLUTIONARY BIOLOGY
BROWN UNIVERSITY
PROVIDENCE, RI 02912

VIJG, JAN, PHD
PROFESSOR
DEPARTMENT OF GENETICS
ALBERT EINSTEIN COLLEGE OF MEDICINE
BRONX, NY 10461

WOLOZIN, BENJAMIN L., PHD, MD
PROFESSOR
DEPARTMENTS OF PHARMACOLOGY AND NEUROLGY
BOSTON UNIVERSITY SCHOOL OF MEDICINE
BOSTON, MA 02118

ZAIDI, MONE, MBBS, PHD, MD
DIRECTOR
DIVISION OF ENDOCRINOLOGY
MOUNT SINAI SCHOOL OF MEDICINE
NEW YORK, NY 10029

**MEMBERS**

AGARWAL, SUDHA, PHD
PROFESSOR
DEPARTMENT OF ORTHOPEDICS
COLLEGE OF MEDICINE
OHIO STATE UNIVERSITY
COLUMBUS, OH 43210

ANDEL, ROSS, PHD
ASSOCIATE PROFESSOR
UNIVERSITY OF SOUTH FLORIDA
SCHOOL OF AGING STUDIES
TAMPA, FL 33620

ANTON, STEPHEN D., PHD
ASSOCIATE PROFESSOR
AGING AND GERIATRIC RESEARCH
UNIVERSITY OF FLORIDA
GAINESVILLE, FL 32611

ARANCIO, OTTAVIO, PHD, MD
ASSOCIATE PROFESSOR
DEPARTMENT OF PATHOLOGY AND CELL BIOLOGY
COLUMBIA UNIVERSITY MEDICAL CENTER
NEW YORK, NY 10032

ARBEEV, KONSTANTIN G., PHD
DEPARTMENT OF SOCIOLOGY
DUKE UNIVERSITY
DURHAM, NC 27708

BANNASCH, DANIKA L., PHD, DVM
MAXINE ADLER ENDOWED CHAIR
DEPARTMENT OF POPULATION HEALTH AND REPRODUCTION
SCHOOL OF VETERINARY MEDICINE
UNIVERSITY OF CALIFORNIA DAVIS
DAVIS, CA 95616

BARGER, STEVEN W., PHD
PROFESSOR
REYNOLDS INSTITUTE ON AGING
UNIVERSITY OF ARKANSAS FOR MEDICAL SCIENCES
LITTLE ROCK, AR 72205

BATTERMAN, STUART A., PHD
PROFESSOR
DEPARTMENT OF ENVIORNMENTAL HEALTH SCIENCES
SCHOOL OF PUBLIC HEALTH
UNIVERSITY OF MICHIGAN
ANN ARBOR, MI 48109-2029

BAUR, JOSEPH A., PHD
ASSISTANT PROFESSOR OF PHYSIOLOGY
DEPARTMENT OF PHYSIOLOGY
PERELMAN SCHOOL OF MEDICINE
UNIVERSITY OF PENNSYLVANIA
PHILADELPHIA, PA 19104

BERNARD, DANIEL J., PHD

PROFESSOR
DEPARTMENT OF PHARMACOLOGY AND THERAPEUTICS
MCGILL UNIVERSITY
MONTREAL, QC H3G 1Y6
CANADA

BLACKER, DEBORAH L., MD, SCD
PROFESSOR
DEPARTMENT OF PSYCHIATRY
MASSACHUSETTS GENERAL HOSPITAL
CHARLESTOWN, MA 02129-2000

BLAU, DAVID M., PHD
PROFESSOR
DEPARTMENT OF ECONOMICS
THE OHIO STATE UNIVERSITY
COLUMBUS, OH 43210

BOND, DALE S., PHD
ASSOCIATE PROFESSOR
WEIGHT CONTROL AND DIABETES RESEARCH CENTER
THE MIRIAM HOSPITAL
BROWN UNIVERSITY
PROVIDENCE, RI 02903

BUCKNER, RANDY L., PHD
PROFESSOR
DEPARTMENT OF PSYCHOLOGY
HARVARD UNIVERSITY
CAMBRIDGE, MA 02138

BUNDORF, MARY KATE, PHD
ASSOCIATE PROFESSOR
DEPARTMENT OF HEALTH RESEARCH AND POLICY
STANFORD UNIVERSITY SCHOOL OF MEDICINE
STANFORD, CA 94305

BROWN-BORG, HOLLY M., PHD
PROFESSOR
DEPARTMENT OF BASIC SCIENCES
SCHOOL OF MEDICINE AND HEALTH SCIENCES
UNIVERSITY OF NORTH DAKOTA
GRAND FORKS, ND 58203

CALOF, ANNE LEIGHTON, PHD
PROFESSOR
DEPARTMENT OF ANATOMY AND NEUROBIOLOGY
COLLEGE OF MEDICINE
UNIVERSITY OF CALIFORNIA, IRVINE
IRVINE, CA 92697

CHATTERJI, SOMNATH, MBBS, MD
WORLD HEALTH ORGANIZATION
HEALTH STATISTICS & INFORMATICS
GENEVA, SWITZERLAND

CHEADLE, JACOB E., PHD
ASSOCIATE PROFESSOR
UNIVERSITY OF NEBRASKA LINCOLN
LINCOLN, NE 68512

CHI, GUANGQING, PHD
ASSOCIATE PROFESSOR
DEPARTMENT OF AGRICULTURAL ECONOMICS, SOCIOLOGY AND EDUCATION
PENNSYLVANIA STATE UNIVERSITY
UNIVERSITY PARK, PA 16802

CHILDS, GWEN V., PHD
PROFESSOR AND CHAIR
DEPARTMENT OF NEUROBIOLOGY AND DEVELOPMENTAL SCIENCES
UNIVERSITY OF ARKANSAS
LITTLE ROCK, AR 72205

CIRRITO, JOHN R., PHD
ASSOCIATE PROFESSOR
DEPARTMENT OF NEUROLOGY
WASHINGTON UNIVERSITY SCHOOL OF MEDICINE
ST. LOUIS, MO 63110

CONNEELY, KAREN, PHD
ASSISTANT PROFESSOR
DEPARTMENT OF HUMAN GENETICS
EMORY UNIVERSITY SCHOOL OF MEDICINE
ATLANTA, GA 30322

CONOVER, CHERYL A., PHD
PROFESSOR
DEPARTMENT OF INTERNAL MEDICINE
MAYO CLINIC COLLEGE OF MEDICINE
ROCHESTER, MN 55905

CURRAN, SARA R., PHD
ASSOCIATE PROFESSOR
DANIEL J. EVANS SCHOOL OF PUBLIC AFFAIRS

UNIVERSITY OF WASHINGTON
SEATTLE, WA 98195

DAVIS, MICHAEL S., DVM, PHD
CHIEF OFFICER
CENTER FOR VETERINARY HEALTH SCIENCES
OKLAHOMA STATE UNIVERSITY
STILLWATER, OK 74078

ELLIOTT, DAWN M., PHD
PROFESSOR AND CHAIR
DEPARTMENT OF BIOMEDICAL ENGINEERING
UNIVERSITY OF DELAWARE
NEWARK, DE 19716

FISCHER, WOLFGANG H., PHD
SENIOR STAFF SCIENTIST
THE CLAYTON FOUNDATION
LABORATORIES FOR PEPTIDE BIOLOGY
THE SALK INSTITUTE
LA JOLLA, CA 92037

FERGUSON, SHAWN, PHD
ASSISTANT PROFESSOR
DEPARTMENT OF CELL BIOLOGY
YALE UNIVERISTY SCHOOL OF MEDICINE
NEW HAVEN, CT 06510

FRISINA, ROBERT D., PHD
PROFESSOR AND BME DIRECTOR
DEPARTMENT OF CHEMICAL
AND BIOMEDICAL ENGINEERING
UNIVERSITY OF SOUTH FLORIDA
TAMPA, FL 33620

GIBSON, GARY J., PHD
HEAD, SECTION OF CELL BIOLOGY
BONE AND JOINT CENTER
HENRY FORD HOSPITAL
DETROIT, MI 48202

GOLDRING, MARY B., PHD
PROFESSOR
CELL AND DEVELOPMENTAL BIOLOGY
WEILL CORNELL MEDICAL COLLEGE
NEW YORK, NY 10021

GRANHOLM-BENTLEY, ANN-CHARLOTTE ESTHER, DDS, PHD
PROFESSOR AND DIRECTOR
KNOEBEL CENTER FOR THE STUDY OF AGINGUNIVERSITY OF DENVER
DENVER, CO 80208

GUTMANN, MYRON P., PHD
DIRECTOR AND PROFESSOR OF HISTORY
UNIVERSITY OF COLORODO
1440 15TH STREE5T
BOULDER, CO 80309

HAMILTON, KARYN L., PHD
PROFESSOR
DEPARTMENT OF HEALTH AND EXERCISE SCIENCE
COLORADO STATE UNIVERSITY
FORT COLLINS, CO 80523

JAMES, BRYAN DAVID, PHD
ASSISTANT PROFESSOR
RUSH ALZHEIMER'S DISEASE CENTER
CHICAGO, IL 60612

JASPER, HEINRICH, PHD
PROFESSOR
THE BUCK INSTITUTE FOR RESEARCH ON AGING
NOVATO, CA 94945

JENNINGS, JULIA, PHD
ASSISTANT PROFESSOR
STATE UNIVERSITY OF NEW YORK AT ALBANY
ALBANY, NY 12202

JOHNSON, DAVID K., PHD
ASSOCIATE PROFESSOR
DEPARTMENT OF PSYCHOLOGY
UNIVERSITY OF KANSAS
LAWRENCE, KS 66045

JOHNSON, MATTHEW WAYNE, PHD
ASSOCIATE PROFESSOR
DEPARTMENT OF PSYCHIATRY
AND BEHAVIORAL SCIENCES
JOHNS HOPKINS UNIVERSITY SCHOOL OF MEDICINE
BALTIMORE, MD 21224

JONES, DAMON, PHD
ASSISTANT PROFESSOR
THE HARRIS SCHOOL OF PUBLIC POLICY STUDIES

UNIVERSITY OF CHICAGO
CHICAGO, IL 60637

KAKAR, SHAM S., PHD
PROFESSOR
DEPARTMENT OF MEDICINE
UNIVERSITY OF LOUISVILLE
LOUISVILLE, KY 40202

KANG, DAVID E., PHD
ASSOCIATE PROFESSOR
DEPARTMENT OF MOLECULAR MEDICINE
BYRD ALZHEIMER'S INSTITUTE
UNIVERSITY OF SOUTH FLORIDA
TAMPA, FL 33613

KASAKOFF, ALICE BEE, PHD
DEPARTMENT OF ANTHROPOLOGY
UNIVERSITY OF SOUTH CAROLINA AT COLUMBIA
COLUMBIA, SC 29208

KAUWE, JOHN SAI KEONG, PHD
ASSOCIATE PROFESSOR
DEPARTMENTS OF BIOLOGY AND NEUROSCIENCE
BRIGHAM YOUNG UNIVERSITY
PROVO, UT 84602

KENNEDY, BRIAN K., PHD
PRESIDENT, CEO AND PROFESSOR
BUCK INSTITUTE FOR RESEARCH ON AGING
NOVATO, CA 94945

KETCHAM, JONATHAN DAVID, PHD
ASSOCIATE PROFESSOR
W.P. CAREY SCHOOL OF BUSINESS
DEPARTMENT OF MARKETING
ARIZONA STATE UNIVERSITY
TEMPE, AZ 85287

KITSIS, RICHARD N., MD
PROFESSOR
DORROS CHAIR IN CARDIOVASCULAR DISEASE
DIRECTOR, WILF FAMILY CARDIOVASCULAR RESEARCH INSTITUTE
ALBERT EINSTEIN COLLEGE OF MEDICINE
BRONX, NY 10461

KOHLER, HANS-PETER, PHD
PROFESSOR
DEPARTMENT OF SOCIOLOGY
UNIVERSITY OF PENNSYLVANIA
PHILADELPHIA, PA 19104

KONDRATOV, ROMAN V., PHD
ASSOCIATE PROFESSOR
DEPARTMENT OF BIOLOGICAL, GEOLOGICAL, AND ENVIRONMENTAL SCIENCES
CLEVELAND STATE UNIVERSITY
CLEVELAND, OH 44115

KRAMER, JOEL H., PSYD
PROFESSOR
DEPARTMENT OF NEUROLOGY
UNIVERSITY OF CALIFORNIA, SAN FRANCISCO
SAN FRANCISCO, CA 94158

KRYSCIO, RICHARD J., PHD
PROFESSOR AND CHAIR
DEPARTMENT OF STATISTICS AND BIOSTATISTICS
UNIVERSITY OF KENTUCKY
LEXINGTON, KY 40536

LA SPADA, ALBERT R., MD, PHD
PROFESSOR
DEPARTMENTS OF PEDIATRICS, CELLULAR AND MOLECULAR MEDICINE AND NEUROSCIENCES
UNIVERSITY OF CALIFORNIA AT SAN DIEGO
LA JOLLA, CA 92093

LEFLER, LEANNE L., PHD, BSN
ASSOCIATE PROFESSOR
DEPARTMENT OF NURSING
COLLEGE OF NURSING
UNIVERSITY OF ARKANSAS FOR MEDICAL SCIENCES
LITTLE ROCK, AR 72205

LEGER, PIERRE
ASSOCIATE PROFESSOR
DIVISION OF HEALTH POLICY AND ADMINISTRATION
SCHOOL OF PUBLIC HEALTH
UNIVERSITY OF ILLINOIS AT CHICAGO
CHICAGO, IL 60612

LOSASSO, ANTHONY T., PHD
PROFESSOR AND SENIOR RESEARCH SCIENTIST
HEALTH POLICY AND ADMINISTRATION
UNIVERSITY OF ILLINOIS AT CHICAGO
CHICAGO, IL 60612

MAO, ZIXU, MD, PHD
PROFESSOR
DEPARTMENT OF PHARMACOLOGY AND NEUROLOGY
EMORY UNIVERSITY SCHOOL OF MEDICINE
ATLANTA, GA 30322

MARTIN, GEORGE M., MD
PROFESSOR
DEPARTMENT OF PATHOLOGY
UNIVERSITY OF WASHINGTON
SEATTLE, WA 98195

MCCARTER, ROGER JOHN, PHD
PROFESSOR
DEPARTMENT OF PHYSIOLOGY
UNIVERSITY OF TEXAS HEALTH SCIENCE CENTER
SAN ANTONIO, TX 78284

MEDVENE, LOUIS J., PHD
PROFESSOR
DEPARTMENT OF PSYCHOLOGY
WICHITA STATE UNIVERSITY
WICHITA, KS 67260

MOODY, JAMES, PHD
PROFESSOR
DEPARTMENT OF SOCIOLOGY
DUKE UNIVERSITY
DURHAM, NC 27708

MURPHY, COLEEN TARA, PHD
PROFESSOR
DEPARTMENT OF MOLECULAR BIOLOGY AND GENOMICS
PRINCETON UNIVERSITY
PRINCETON, NJ 08544

NAVARATNAM, DHASAKUMAR S., MD, PHD
ASSOCIATE PROFESSOR
DEPARTMENT OF NEUROLOGY AND NEUROBIOLOGY
YALE UNIVERSITY
NEW HAVEN, CT 06510

NOSAL, KATHLEEN, PHD
ASSISTANT PROFESSOR
DEPARTMENT OF ECONOMICS
UNIVERSITY OF MANNHEIM
MANNHEIM 68161
GERMANY

OLSEN, RANDALL J., PHD
PROFESSOR
DEPARTMENT OF ECONOMICS
THE OHIO STATE UNIVERSITY
COLUMBUS, OH 43221

PAGE, RODNEY LEE, DVM
DIRECTOR
DEPARTMENT OF CLINICAL SCIENCES
COLORADO STATE UNIVERSITY
DENVER, CO 80208

PETERS-LIBEU, CLARE A., PHD
STAFF SCIENTIST
BUCK INSTITUTE FOR RESEARCH ON AGING
NOVATO, CA 94945

PEELLE, JONATHAN E., PHD
ASSISTANT PROFESSOR
WASHINGTON UNIVERSITY
SAINT LUIS, MO 63110

SALVY, SARAH-JEANNE, PHD
ASSOCIATE PROFESSOR
CHAN DIVISION OF OCCUPATIONAL SCIENCE AND OCCUPATIONAL THERAPY
UNIVERSITY OF SOUTHERN CALIFORNIA
LOS ANGELES, CA 90089

SCHAFFER, DAVID V., PHD
PROFESSOR
DEPARTMENT OF CHEMICAL ENGINEERING
UNIVERSITY OF CALIFORNIA, BERKELEY
BERKELEY, CA 94720

SCHNEIDER, JULIE A., MD
PROFESSOR AND ASSOCIATE DIRECTOR
DEPARTMENT OF PATHOLOGY
RUSH ALZHEIMER'S DISEASE CENTER
RUSH UNIVERSITY MEDICAL CENTER
CHICAGO, IL 60612

SELL, CHRISTIAN, PHD

ASSOCIATE PROFESSOR
DEPARTMENT OF PATHOLOGY
COLLEGE OF MEDICINE
DREXEL UNIVERSITY
PHILADELPHIA, PA 19102

SHELINE, YVETTE I., MD
PROFESSOR AND DIRECTOR
DEPARTMENT OF PSYCHIATRY
CENTER FOR THE NEUROMODULATION IN DEPRESSION AND STRESS
UNIVERSITY OF PENNSYLVANIA SCHOOL OF MEDICINE
PHILADELPHIA, PA 19104

SIMMONS, DWAYNE D., PHD
PROFESSOR AND DIRECTOR OF MARC/BRIDGES
DEPARTMENT OF INTEGRATIVE BIOLOGY AND PHYSIOLOGY
UNIVERSITY OF CALIFORNIA, LOS ANGELES
LOS ANGELES, CA 90095

SUBRAMANIAN, SUJHA, PHD
SENIOR HEALTH ECONOMIST
DIVISION OF HEALTH ECONOMICS RESEARCH
RESEARCH TRIANGLE INSTITUTE
WALTHAM, MA 02452-8414

STAFFORD, FRANK PETER, PHD
PROFESSOR OF ECONOMICS
DEPARTMENT OF ECONOMICS
UNIVERSITY OF MICHIGAN
ANN ARBOR, MI 48106

SWARTZ, ANN M., PHD
PROFESSOR AND CHAIR
DEPARTMENT OF KINESIOLOGY
EXERCISE SCIENCE & HEALTH PROMOTION
COLLEGE OF HEALTH SCIENCES
UNIVERSITY OF WISCONSIN-MILWAUKEE
MILWAUKEE, WI 53201-0413

TEMPLE, SALLY, PHD
PROFESSOR
NEW YORK NEURAL STEM INSTITUTE
RENSSELAER, NY 12144

THOMPSON, WINSTON E., PHD
PROFESSOR
DEPARTMENT OF PHYSIOLOGY
MOREHOUSE SCHOOL OF MEDICINE
ATLANTA, GA 30310

THYFAULT, JOHN P., PHD
ASSOCIATE PROFESSOR
DEPARTMENT OF MOLECULAR INTEGRATIVE PHYSIOLOGY
UNIVERSITY OF KANSAS MEDICAL CENTER
KANSAS CITY, KS 66160

TRAVISON, THOMAS GLENN, PHD
SENIOR SCIENTIST AND DIRECTOR
INSTITUTE FOR AGING RESEARCH
DIVISION OF GERONTOLOGY
HARVARD MEDICAL SCHOOL
BOSTON, MA 02131

TRUSHINA, EUGENIA, PHD
ASSOCIATE PROFESSOR
DEPARTMENT OF NEUROLOGY AND PHARMACOLOGY
MAYO CLINIC COLLEGE OF MEDICINE
ROCHESTER, MN 55905

VARELA, MAURICIO, PHD
ASSISTANT PROFESSOR
DEPARTMENT OF ECONOMICS
UNIVERSITY OF ARIZONA
TUCSON, AZ 85721

VILLEDA, SAUL A., PHD
ASSISTANT PROFESSOR
UNIVERSITY OF CALIFORNIA SAN FRANCISCO
SAN FRANCISCO, CA 94143

WAIDMANN, TIMOTHY A., PHD
SENIOR RESEARCH ASSOCIATE
HEALTH POLICY CENTER
URBAN INSTITUTE
WASHINGTON, DC 20037

WALKER, WILLIAM H., PHD
ASSOCIATE PROFESSOR
DEPARTMENT OF CELL BIOLOGY AND PHYSIOLOGY
UNIVERSITY OF PITTSBURGH
PITTSBURGH, PA 15261

WANG, HONGMEI, PHD

UNIVERSITY OF NEBRASKA MEDICAL CENTER
OMAHA, NE 68198

WELSH-BOHMER, KATHLEEN ANNE, PHD
PROFESSOR AND DIRECTOR
DEPARTMENTS OF PSYCHOLOGY AND NEUROLOGY
ALZHEIMER'S DISEASE CENTER
DUKE UNIVERSITY
DURHAM, NC 27705

WEINSTEIN, MAXINE A., PHD
DISTINGUISHED PROFESSOR
GRADUATE SCHOOL OF ARTS AND SCIENCES
CENTER FOR POPULATION AND HEALTH
GEORGETOWN UNIVERSITY
WASHINGTON, DC 20057

WESTENDORF, JENNIFER J., PHD
MARGARET AMINI PROFESSOR
DEPARTMENT OF ORTHOPEDICS, BIOCHEMISTRY AND MOLECULAR BIOLOGY
MAYO CLINIC
ROCHESTER, MN 55905

WILKER, ELISSA, SCD
ASSISTANT PROFESSOR OF MEDICINE
DEPARTMENT OF MEDICINE AND EPEDEMIOLOGY
HARVARD MEDICAL SCHOOL
BOSTON, MA 02215

WINGFIELD, ARTHUR, PHD
PROFESSOR
DEPARTMENT OF PSYCHOLOGY AND NEUROSCIENCE
DIRECTOR, VOLEN NATIONAL CENTER FOR COMPLEX SYSTEMS
BRANDEIS UNIVERSITY
WALTHAM, MA 02454

YUE, ZHENYU, PHD
PROFESSOR
DEPARTMENT OF NEUROLOGY AND NEUROSCIENCE
ICAHN SCHOOL OF MEDICINE AT MOUNT SINAI
NEW YORK, NY 10029

ZAGHENI, EMILIO, PHD
ASSISTANT PROFESSOR
DEPARTMENT OF SOCIOLOGY
UNIVERSITY OF WASHINGTON
SEATTLE, WA 98195

ZHANG, MIN, PHD, MD
ASSOCIATE PROFESSOR
DEPARTMENT OF STATISTICS
PURDUE UNIVERSITY
WEST LAFAYETTE, IN 47907

ZHANG, QI, PHD
ASSISTANT PROFESSOR
DEPARTMENT OF PHARMACOLOGY
VANDERBILT UNIVERSITY MEDICAL CENTER
NASHVILLE, TN 37232

ZHENG, HUI, PHD
PROFESSOR AND DIRECTOR
HUFFINGTON CENTER ON AGING
BAYLOR COLLEGE OF MEDICINE
HOUSTON, TX 77030

**SCIENTIFIC REVIEW OFFICER**

BISSONETTE, GREG, PHD
SCIENTIFIC REVIEW BRANCH
NATIONAL INSTITUTE ON AGING
NATIONAL INSTITUTES OF HEALTH
BETHESDA, MD 20892

FIRTH, KIMBERLY, PHD
SCIENTIFIC REVIEW BRANCH
NATIONAL INSTITUTE ON AGING
NATIONAL INSTITUTES OF HEALTH
BETHESDA, MD 20892

GRIMALDI, MAURIZIO, MD, PHD
SCIENTIFIC REVIEW OFFICER
NATIONAL INSTITUTE ON AGING
NATIONAL INSTITUTES OF HEALTH
BETHESDA, MD 20892

MARKOWSKA, ALICJA L., PHD, DSC
SCIENTIFIC REVIEW BRANCH
NATIONAL INSTITUTE ON AGING
NATIONAL INSTITUTES OF HEALTH
BETHESDA, MD 20982

MIKHAIL, ISIS S., MD, MPH, DRPH
SCIENTIFIC REVIEW BRANCH

NATIONAL INSTITUTE ON AGING
NATIONAL INSTITUTES OF HEALTH
BETHESDA, MD 20892

MOTEN, CARMEN, PHD, MPH
SCIENTIFIC REVIEW BRANCH
NATIONAL INSTITUTE ON AGING
NATIONAL INSTITUTES OF HEALTH
BETHESDA, MD 20892

NAKHAI, BITA, PHD
SCIENTIFIC REVIEW BRANCH
NATIONAL INSTITUTE ON AGING
NATIONAL INSTITUTES OF HEALTH
BETHESDA, MD 20814

PARSADANIAN, ALEXANDER, PHD
SCIENTIFIC REVIEW OFFICER
SCIENTIFIC REVIEW BRANCH
NATIONAL INSTITUTE ON AGING
NATIONAL INSTITUTES OF HEALTH
BETHESDA, MD 20892

PRASAD, NIJAGUNA, MS, PHD
SCIENTIFIC REVIEW OFFICER
SCIENTIFIC REVIEW BRANCH
NATIONAL INSTITUTE ON AGING
NATIONAL INSTITUTES OF HEALTH
BETHESDA, MD 20892

**EXTRAMURAL SUPPORT ASSISTANT**

GLENN, JENNIFER N
EXTRAMURAL SUPPORT ASSISTANT
SCIENTIFIC REVIEW BRANCH
NATIONAL INSTITUTE ON AGING
NATIONAL INSTITUTES OF HEALTH
BETHESDA, MD 20894

WILSON, SHAYLA K
EXTRAMURAL SUPPORT ASSISTANT
SCIENTIFIC REVIEW BRANCH
NATIONAL INSTITUTE ON AGING
NATIONAL INSTITUTE OF HEALTH
BETHESDA, MD 20892

WOODEN, EZELLE III
EXTRAMURAL SUPPORT ASSISTANT
SCIENTIFIC REVIEW BRANCH
NATIONAL INSTITUTE ON AGING
NATIONAL INSTITUTES OF HEALTH
BETHESDA, MD 20892

**PROGRAM REPRESENTATIVE**

BHATTACHARYYA, PARTHA, PHD
PROGRAM DIRECTOR
DIVISION OF BEHAVIORAL AND SOCIAL RESEARCH
NATIONAL INSTITUTE ON AGING
NATIONAL INSTITUTES OF HEALTH
BETHESDA, MD 20892

FRIDELL, YIH-WOEI
DIVISION OF AGING BIOLOGY
NATIONAL INSTITUTE ON AGING
NATIONAL INSTITUTES OF HEALTH
BETHESDA, MD 20892

FULDNER, REBECCA A., PHD
HEALTH SCIENCE ADMINISTRATOR
BIOLOGY OF AGING PROGRAM
NATIONAL INSTITUTE ON AGING
BETHESDA, MD 20814

HSIAO, JOHN, MD
HEALTH SCIENTIST ADMINISTRATOR
DIVISION OF NEUROSCIENCE
NATIONAL INSTITUTE ON AGING
NATIONAL INSTITUTES OF HEALTH
BETHESDA, MD 20892

JOSEPH, LYNDON J O, PHD
ROMASHKAN, SERGEI, MD, PHD
BRANCH CHIEF
HEALTH SCIENTIST ADMINISTRATOR
GERIATRICS AND CLINICAL GERONTOLOGY
NATIONAL INSTITUTE ON AGING
NATIONAL INSTITUTES OF HEALTH
BETHESDA, MD 20892

KOHANSKI, RONALD A., PHD
DEPUTY DIRECTOR
BIOLOGY OF AGING PROGRAM
NATIONAL INSTITUTE ON AGING
BETHESDA, MD 20814

PATMIOS, GEORGEANNE E.,
PROGRAM OFFICER
DIVISION OF BEHAVIORAL AND SOCIAL RESEARCH
NATIONAL INSTITUTE ON AGING
NATIONAL INSTITUTES OF HEALTH

BETHESDA, MD 20892

ROMASHKAN, SERGEI, MD, PHD
BRANCH CHIEF
HEALTH SCIENCE ADMINISTRATOR
DIVISION OF GERIATRICS AND CLINICAL GERONTOLOGY
NATIONAL INSTITUTE ON AGING
NATIONAL INSTITUTES OF HEALTH
BETHESDA, MD 20893

WILLIAMS, JOHN PHILIP, PHD
HEALTH SCIENCE ADMINISTRATOR
NATIONAL INSTITUTE OF HEALTH
NATIONAL INSTITUTE OF AGING
DIVISION OF AGING BIOLOGY
BETHESDA, MD 20892

WISE, BRADLEY C., PHD
CHIEF, HEALTH SCIENTIST ADMINISTRATOR
DIVISION OF NEUROSCIENCE
NATIONAL INSTITUTE ON AGING
NATIONAL INSTITUTES OF HEALTH
BETHESDA, MD 20892

YANG, AUSTIN JYAN-YU
HEALTH SCIENTIST ADMINISTRATOR
DIVISION OF NEUROSCIENCE
NATIONAL INSTITUTE ON AGING
BETHESDA, MD 20892

---

Footnotes for 1 P01 AG052352-01A1; PI Name: LACROIX, ANDREA Z.

NIH has modified its policy regarding the receipt of resubmissions (amended applications). See Guide Notice NOT-OD-14-074 at http://grants.nih.gov/grants/guide/notice-files/NOT-OD-14-074.html. The impact/priority score is calculated after discussion of an application by averaging the overall scores (1-9) given by all voting reviewers on the committee and multiplying by 10. The criterion scores are submitted prior to the meeting by the individual reviewers assigned to an application, and are not discussed specifically at the review meeting or calculated into the overall impact score. Some applications also receive a percentile ranking. For details on the review process, see http://grants.nih.gov/grants/peer_review_process.htm#scoring.

MEETING ROSTER
National Institute on Aging Special Emphasis Panel

NATIONAL INSTITUTE ON AGING
Second Stage P01 Review
ZAG1 ZIJ-7 (J1)
11/21/2016


CHAIRPERSON(S)

MELTZER, DAVID O, MD, PHD
PROFESSOR
DEPARTMENT OF MEDICINE
CHIEF, SECTION OF HOSPITAL MEDICINE
DIRECTOR, CENTER FOR HEALTH AND THE SOCIAL SCS.
UNIVERSITY OF CHICAGO
CHICAGO, IL 60637

MEMBERS

BICKFORD, PAULA C, PHD
DISTINGUISHED USF HEALTH PROFESSOR
DEPARTMENT OF NEUROSURGERY
AND BRAIN REPAIR
USF MORSANI COM
MDC-78
TAMPA, FL 33612

CAMPISI, JUDITH, PHD
PROFESSOR
BUCK INSTITUTE FOR AGE RESEARCH
LABORATORY OF CELLULAR AND
MOLECULAR BIOLOGY OF AGING
NOVATO, CA 94945

CARSTENSEN, LAURA L, PHD
PROFESSOR
THE FAIRLEIGH S. DICKINSON JR. PROFESSOR IN
PUBLIC POLICY
DIRECTOR, STANFORD CENTER ON LONGEVITY
STANFORD UNIVERSITY
STANFORD, CA 94305

DE LEON, MONY J., EDD
PROFESSOR OF PSYCHIATRY
DEPARTMENT OF PSYCHIATRY
SCHOOL OF MEDICINE
NEW YORK UNIVERSITY
NEW YORK, NY 10016

KRITCHEVSKY, STEPHEN B., PHD
DIRECTOR
STRICHT CENTER ON AGING
DEPARTMENT OF INTERNAL MEDICINE
WAKE FOREST UNIVERSITY
WINSTON-SALEM, NC 27157

KUCHEL, GEORGE A, MD
DIRECTOR, UCONN CENTER ON AGING
DEPARTMENT OF MEDICINE
CENTER OF AGING
UNIVERSITY OF CONNECTICUT HEALTH CENTER
FARMINGTON, CT 06030

RICHARDSON, ARLAN G., PHD
PROFESSOR
UNIVERSITY OF OKLAHOMA
ROCA/GERIATRIC MEDICINE
OKLAHOMA CITY, OK 73104

ROSENE, DOUGLAS L, PHD
PROFESSOR
DEPARTMENT OF ANTATOMY AND NEUROBIOLGY
BOSTON UNIVERSITY SCHOOL OF MEDICINE
BOSTON, MA 02118

SIEBER, FREDERICK E, MD
PROFESSOR
DEPARTMENT OF ANESTHESIOLOGY/
CRITICAL CARE MEDICINE
JOHNS HOPKINS UNIVERSITY
BALTIMORE, MD 21224

SMITH, JAMES PATRICK, PHD
NEPHROLOGIST AND MEDICAL OFFICER
RAND CORPORATION
DEPT OF LABOR AND POPULATION
SANTA MONICA, CA 90407-2138

VAN ELDIK, LINDA J, PHD
PROFESSOR AND DIRECTOR
SANDERS-BROWN CENTER ON AGING
UNIVERSITY OF KENTUCKY
LEXINGTON, KY 40536

SCIENTIFIC REVIEW OFFICER

VEMURI, RAMESH, PHD
CHIEF, SCIENTIFIC REVIEW BRANCH
NATIONAL INSTITUTE ON AGING
NATIONAL INSTITUTES OF HEALTH
BETHESDA, MD 20892

EXTRAMURAL SUPPORT ASSISTANT

WILSON, SHAYLA K
EXTRAMURAL SUPPORT ASSISTANT
NATIONAL INSTITUTE ON AGING
NATIONAL INSTITUTE OF HEALTH
BETHESDA, MD 20892

Consultants are required to absent themselves from the room during the review of any application if their presence would constitute or appear to constitute a conflict of interest.
